# Rifamycin-Related Polyketides from a Marine-Derived Bacterium *Salinispora arenicola* and Their Cytotoxic Activity

**DOI:** 10.3390/md21090494

**Published:** 2023-09-15

**Authors:** Cao Van Anh, Jong Soon Kang, Jeong-Wook Yang, Joo-Hee Kwon, Chang-Su Heo, Hwa-Sun Lee, Hee Jae Shin

**Affiliations:** 1 Marine Natural Products Chemistry Laboratory, Korea Institute of Ocean Science and Technology, 385 Haeyang-ro, Yeongdo-gu, Busan 49111, Republic of Korea; caovananh12a1@gmail.com (C.V.A.); science30@kiost.ac.kr (C.-S.H.); hwasunlee@kiost.ac.kr (H.-S.L.); 2 Laboratory Animal Resource Center, Korea Research Institute of Bioscience and Biotechnology, 30 Yeongudanjiro, Cheongju 28116, Republic of Korea; kanjon@kribb.re.kr (J.S.K.); z7v8@kribb.re.kr (J.-W.Y.); juhee@kribb.re.kr (J.-H.K.); 3 Department of Marine Biotechnology, University of Science and Technology (UST), 217 Gajungro, Yuseong-gu, Daejeon 34113, Republic of Korea

**Keywords:** *Salinispora arenicola*, polyketides, rifamycin, saliniketal, cytotoxicity

## Abstract

Eight rifamycin-related polyketides were isolated from the culture broth of a marine-derived bacterium *Salinispora arenicola*, including five known (**2**–**5** and **8**) and three new derivatives (**1**, **6**, and **7**). The structures of the new compounds were determined by means of spectroscopic methods (HRESIMS and 1D, 2D NMR) and a comparison of their experimental data with those previously reported in the literature. The isolated compounds were evaluated for their cytotoxicity against one normal, six solid, and seven blood cancer cell lines and **1** showed moderate activity against all the tested cell lines with GI_50_ values ranging from 2.36 to 9.96 µM.

## 1. Introduction

The marine environment covers approximately 70% of planet Earth and hosts a huge biodiversity due to its unique living conditions with high pressure, high salinity, and low oxygen supply [1]. Actinomycetes are the largest reservoir of microbial natural products with interesting bioactivities and have been useful in diverse applications in agriculture and medicine [2]. As an alternative source to the conventionally used terrestrial microorganisms, actinomycetes are ubiquitous in marine environments and are considered an appealing source of novel structures for new drug discovery [3]. The genus *Salinispora* was reported as the first obligate marine actinomycetal genus. Until now, three closely related species from this genus, including *Salinispora tropica*, *S. arenicola*, and *S. pacifica*, have been identified [4]. Members of this genus were commonly found in various substrates in marine environments, including tropical and subtropical sediments, tropical sponges, ascidians, and seaweeds [5]. Although their discovery was relatively late, more than 50 secondary metabolites with a broad range of chemical scaffolds, such as salinosporamides, rifamycins, lomaiviticins, cyclomarins, and salinaphthoquinones, as well as many inspired muta- and semi-synthesis structures, have been reported from this genus [6]. Among the reported compounds, salinosporamide A showed significant activity against various types of cancer and has now entered clinical studies [7]. Based on the unique living conditions and diverse biosynthetic abilities, the genus is considered an emerging source of leading structures for future drug discovery.

Rifamycins are a group of antibiotics produced by several actinomycetes, such as *Amycolatopsis*, *Streptomyces*, *Micormonospora*, *and Salinispora* [8,9,10,11]. The rifamycin group, including classic rifamycin drugs and their semi-synthesized derivatives, is widely used in clinical practice for the treatment of tuberculosis [12]. The structures of rifamycins are quite unique and consist of an aromatic moiety bridged by an aliphatic chain. The aromatic part can be a naphthalene or naphthoquinone ring, and the aliphatic chain is called an ansa-chain [13]. The biosynthesis of rifamycins has been well studied, and their structures were confirmed by X-ray crystallography and total synthesis research [14]. To date, the absolute configuration of the ansa-chain was reported to be identical in all rifamycin derivatives [15].

**Figure 1:**
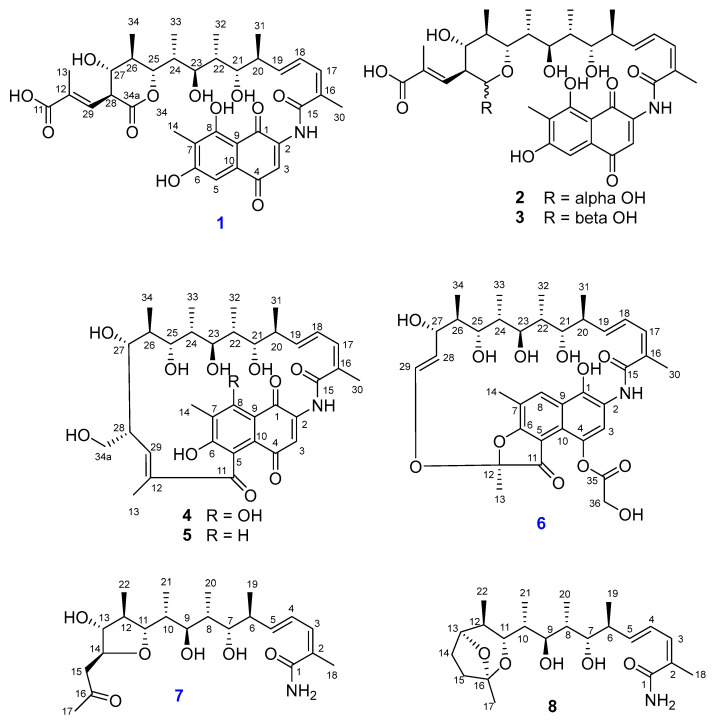
Structures of **1**–**8** isolated from *Salinispora arenicola* 225DD-027.

As part of our ongoing research on natural products produced by actinomycetes near Dokdo island, Republic of Korea, the strain 225DD-027 was isolated from a sediment sample and was identified as *Salinispora arenicola* by 16S rRNA gene sequence analysis. The strain produced orange pigments, which were primarily speculated as rifamycin analogs from a literature review. In a small culture (1 L), the production of pigments was relatively low, and it was insufficient for structural elucidation. Therefore, a mass culture was conducted to obtain a sufficient amount and to determine the structures and biological properties of the pigments. As a result, eight rifamycin-related polyketides, including three new compounds (**1**, **6**, and **7**, Figure 1), were isolated, and the details of their isolation, structure determination, and bioactivities are described in this paper. 

## 2. Results and Discussion

**Figure 2:**
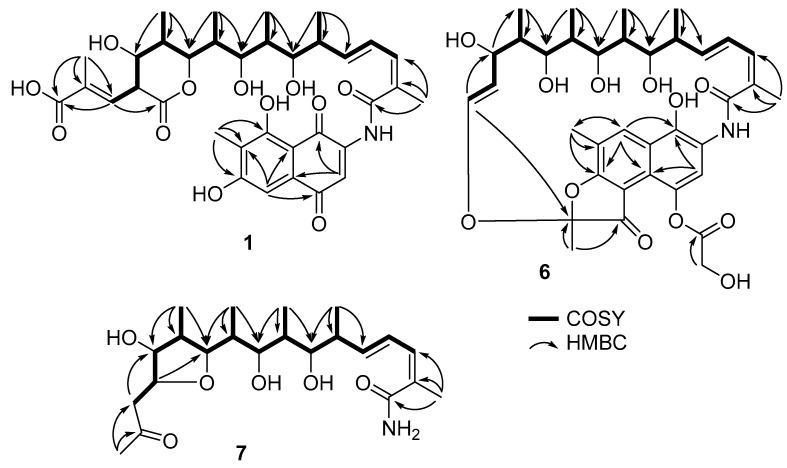
Key ^1^H-^1^H COSY and HMBC correlations for **1**, **6**, and **7**.

**Figure 3:**
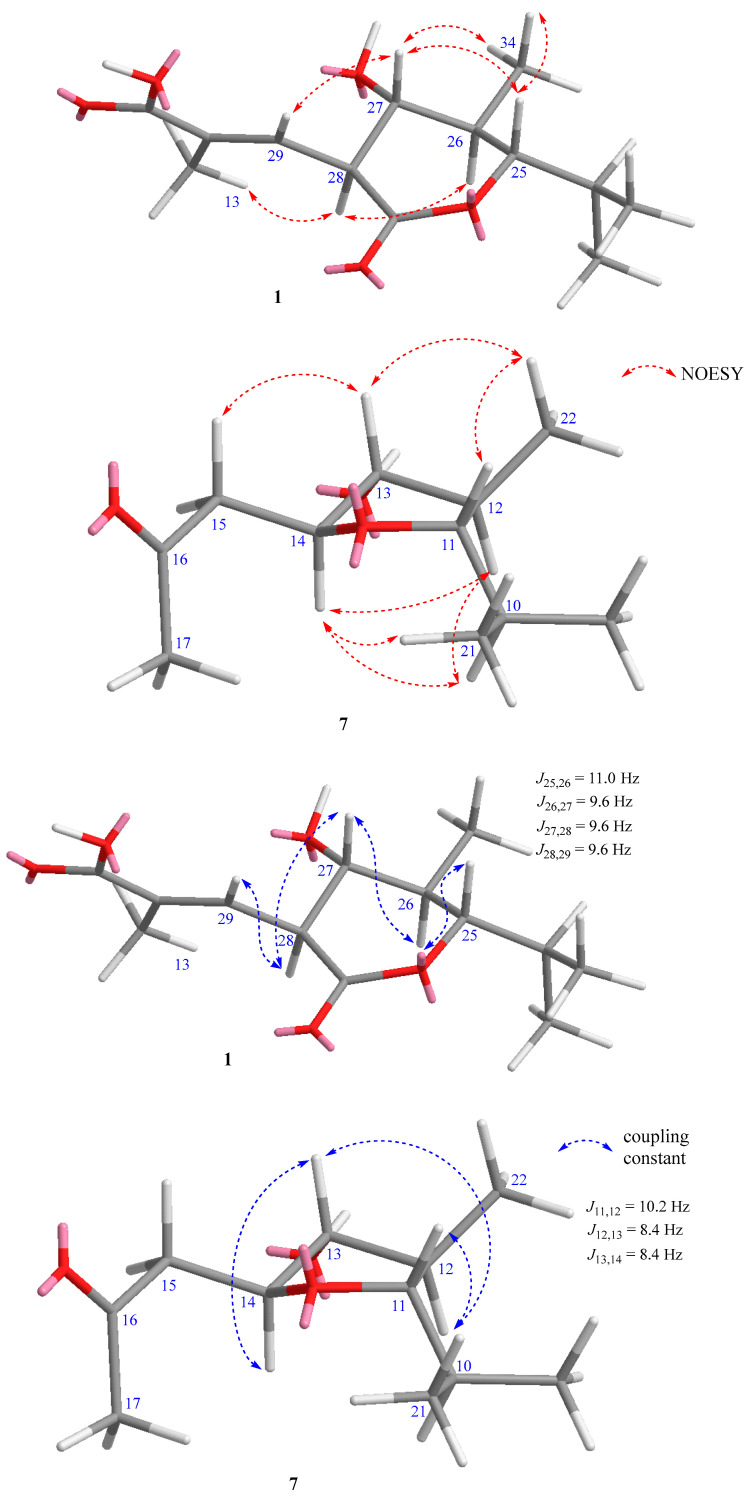
Key NOESY correlations and coupling constants for the lactone ring of **1** and tetrahydrofuran ring of **7**.

Compound **1** was isolated as an orange powder. The molecular formula of **1** was determined as C_35_H_43_NO_12_ by HRESIMS data (*m*/*z* 670.2852, [M + H]^+^, calcd for [C_35_H_44_NO_12_]^+^ 670.2858, −0.9 ppm). The ^1^H NMR spectrum of **1** (Table 1) revealed the presence of two singlet aromatic protons at *δ*_H_ 7.63 (H-3) and 7.05 (H-5); four olefinic protons at *δ*_H_ 6.82 (dd, *J* = 15.0, 11.2 Hz, H-18), 6.68 (dd, *J* = 9.6 and 1.2 Hz, H-29), 6.48 (d, *J* = 11.1 Hz, H-17), and 6.03 (dd, *J* = 15.1, 8.0 Hz, H-19); four oxygenated methines at *δ*_H_ 4.60 (d, *J* = 11.0 Hz, H-25), 3.76 (d, *J* = 9.8 Hz, H-21), 3.65 (dd, *J* = 10.1, 2.6 Hz, H-23), and 3.55 (t, *J* = 9.6 Hz, H-27); five methines at *δ*_H_ 3.45 (t, *J* = 9.6 Hz, H-28), 2.44 (m, H-20), 2.07 (m, H-24), 1.94 (m, H-22), and 1.93 (m, H-26); and seven methyl groups at *δ*_H_ 2.11 (s, H_3_-14), 2.09 (s, H_3_-30), 1.91 (d, *J* = 1.3 Hz, H_3_-13), 1.10 (d, *J* = 7.0 Hz, H_3_-32), 1.08 (d, *J* = 6.5 Hz, H_3_-34), 1.01 (d, *J* = 6.8 Hz, H_3_-31), and 0.89 (d, *J* = 6.9 Hz, H_3_-33). The ^13^C and HSQC data of **1** revealed 35 signals of two ketone carbonyls at *δ*_C_ 186.5 (C-4) and 183.8 (C-1); three carboxyl groups at *δ*_C_ 173.0 (C-34a), 171.4 (C-11), and 169.9 (C-15); two oxygenated sp^2^ carbons at *δ*_C_ 165.1 (C-8) and 163.8 (C-6); twelve sp^2^ carbons at *δ*_C_ 108.2-145.7; four oxygenated methines at *δ*_C_ 74.5-83.1; five methines at *δ*_C_ 35.0-51.6; and seven methyls at *δ*_C_ 8.0-20.5. The ^1^H and ^13^C NMR data of **1** were almost identical to those of the isolated compound, 34a-*α*-rifamycin W-M1-hemiacetal (**2**). The only difference was the chemical shift of C-34a (*δ*_C-34a_ 173.0 for **1**, and *δ*_C-34a_ 94.5, *δ*_H-34a_ 5.08 for **2**), indicating the hemiacetal at C-34a of **2** was oxidized to a carbonyl in **1**, which was re-confirmed by HRESIMS data of **1** with one less degree of unsaturation than that of **2**. The presence of a lactone ring between C-25 and C-34a of **1** was also confirmed by the significant downfield chemical shift of C-25 in **1** compared to those of **2** (*δ*_C-25_ 83.1 and *δ*_H-25_ 4.60 for **1**, and *δ*_C-25_ 72.7, *δ*_H-25_ 4.21 for **2**). Further detailed analysis of 2D NMR data (Figure 2) determined the planar structure of **1**, as shown in Figure 1. The NOESY correlation from H-17 to H_3_-30 indicated the *Z*-configuration of *∆*^16,17^, and the large coupling constant of H-18 and H-19 (*J* = 15.0 Hz) confirmed the *E*-configuration of *∆*^18,19^. The NOESY correlations of H-27/H_3_-29, H-27/H_3_-34, and H-25/H_3_-34 indicated that H-25, H-27, H_3_-29, and H_3_-34 were located on the same face of the lactone ring. The large coupling constants of H-27/H-26 and H-27/H-28 (*J* = 9.6 Hz) confirmed that H-26 and H-28 were located on the opposite face from H-27 (Figure 3). Thus, the relative configuration of the lactone ring was determined to be the same as those of co-isolated compounds (**2** and **3**). The absolute configuration of the ansa-chain was proposed to be the same as that of other rifamycin derivatives by considering their biosynthetic relationship, NMR data, and coupling constants of **1** and **2**. Thus, the structure of **1** was determined, and **1** was given the trivial name of salinisporamycin C. 

Compound **6** was isolated as an orange powder. The molecular formula of **6** was determined to be C_36_H_45_NO_12_ by HRESIMS data (*m*/*z* 706.2809, [M + Na]^+^_,_ calcd for [C_36_H_45_NO_12_Na]^+^ 706.2834, −3.5 ppm). The ^1^H NMR spectrum showed the presence of two singlet aromatic protons at *δ*_H_ 7.21 (H-3) and 8.61 (H-8); five olefinic protons at *δ*_H_ 6.30 (d, *J* = 10.7 Hz, H-17), 6.43 (dd, *J* = 15.4 and 11.2 Hz, H-18), 5.98 (dd, *J* = 15.5 and 5.7 Hz, H-19), 5.22 (dd, *J* = 12.4 and 6.0 Hz, H-28), and 6.19 (d, *J* = 12.5 Hz, H-29); four oxygenated methines at *δ*_H_ 3.95 (d, *J* = 9.4 Hz, H-21), 3.43 (d, *J* = 10.3 Hz, H-23), 3.62 (d, *J* = 10.6 Hz, H-25), and 4.38 (d, *J* = 5.6 Hz, H-27); four methines at *δ*_H_ 2.35 (m, H-20), 1.79 (m, H-22), 1.35 (m, H-24), and 1.06 (m, H-26); oxygenated methylenes at *δ*_H_ 4.70 and 4.56 (d, *J* = 16.9 Hz, H-36a and H-36b); and seven methyl groups at *δ*_H_ 1.68 (s, H_3_-13), 2.49 (s, H_3_-14), 2.07 (s, H_3_-30), 0.92 (d, *J* = 6.9 Hz, H_3_-31), 1.03 (d, *J* = 6.9 Hz, H_3_-32), 0.60 (d, *J* = 6.8 Hz, H_3_-33), and -0.64 (d, *J* = 6.7 Hz, H_3_-34). The ^13^C and HSQC spectra revealed signals of 36 carbons belonging to a ketone at *δ*_C_ 197.6 (C-11); two carbonyls at *δ*_C_ 172.3 (C-15) and 174.0 (C-35); sixteen sp^2^ carbons at *δ*_C_ 112.2–175.5; a hemiketal at *δ*_C_ 108.5 (C-12); four oxygenated methines at *δ*_C_ 68.5–78.3; an oxygenated methylene at *δ*_C_ 62.1 (C-36); four methines at *δ*_C_ 34.5–41.8; and seven methyls at *δ*_C_ 8.8–21.5. The continuous ^1^H-^1^H COSY correlations from H-17 to H-29 via H_3_-31, H_3_-32, H_3_-33, and H_3_-34, as well as the HMBC correlations from H_3_-30 to C-15, C-16, and C-17, established the structure of an ansa-chain. The HMBC correlations from H_3_-14 to C-6, C-7, and C-8; from H-3 to C-1 and C-10; from H-8 to C-1, C-6, C-10, and C-14 established a naphthalene ring with three hydroxy groups at the C-1, C-4, and C-6 positions. The HMBC correlations from H-29 to C-12, H_3_-13 to C-11, and C-12 confirmed the structure of a furan ring and the connection of the ansa-chain with the aromatic moiety via an ether bond between C-29 and C-12. Further, the HMBC correlations from H-36_a,b_ to C-35 determined the presence of glycolic acid. By comparison with data reported in the literature, the structure of **6** was determined as 8-deoxy-25-desacetyl-27-demethyl-rifamycin L [16] and named salinisporamycin D.

Compound **7** was isolated as a colorless solid. The molecular formula of **7** was determined as C_22_H_37_NO_6_ by HRESIMS data (*m*/*z* 434.2496, [M + Na]^+^_,_ calcd for [C_22_H_37_NO_6_Na]^+^ 434.2513, −3.9 ppm), five degrees of unsaturation. The ^1^H NMR spectrum of **7** (Table 2) revealed the presence of three olefinic protons at *δ*_H_ 6.17 (d, *J* = 11.1 Hz, H-3), 6.58 (dd, *J* = 15.2 and 11.1 Hz, H-4), and 5.78 (d, *J* = 15.2 and 8.3 Hz, H-5); five oxygenated methines at *δ*_H_ 3.46–4.05; four methines at *δ*_H_ 1.81–2.34; a methylene group at *δ*_H_ 2.63 and 2.75 (H-15a and H-15b); and six methyls at *δ*_H_ 0.95–2.18. The ^13^C and HSQC NMR spectra demonstrated the presence of 22 carbons, including a ketocarbonyl at *δ*_C_ 210.3 (C-16); a carboxyl at *δ*_C_ 175.1 (C-1); four olefinic carbons at *δ*_C_ 128.3–142.1; five oxygenated methines at *δ*_C_ 75.6–83.5; four methines at *δ*_C_ 36.3–44.0; a methylene at *δ*_C_ 48.5 (C-15); and six methyls at *δ*_C_ 10.6–30.6. The ^1^H and ^13^C NMR data of **7** were similar to those of the co-isolated known compound, saliniketal A (**8**). The significant downfield chemical shifts of C-11 (*δ*_C_ 83.5), C-13 (*δ*_C_ 82.7), and C-14 (*δ*_C_ 80.8), and the HMBC correlation from H-14 (*δ*_H_ 3.95) to C-11 confirmed the partial structure of a tetrahydrofuran ring between C-11 and C-14. The NOESY correlation between H-11, H_3_-22, H-13, and H-15a,b indicated their co-facial relationship, and that of H-14/H-12 indicated that H-14 and H-12 were located on the opposite face of the tetrahydrofuran ring. Saliniketals A and B are bicyclic polyketides isolated from a *Salininispora arenicola* strain [15]. Their structures were elucidated by the 2D NMR techniques and were confirmed by total synthesis studies [8]. Saliniketals shared a partially similar structure with the ansa-chain of rifamycin derivatives isolated from the same strain. The biosynthesis studies also confirmed this relationship, and saliniketals and rifamycin derivatives shared the same absolute configuration [9]. By comparing ^1^H and ^13^C NMR data and the experimental ECD spectrum of **7** with those of saliniketal A (**8**) and considering their biosynthetic relationship, the absolute configuration of **7** was proposed to be the same as that of saliniketal A (**8**) and named salinifuran A. 

Structures of known compounds were identified as 34a-*α*-rifamycin W-M1-hemiacetal (**2**), 34a-*β*-rifamycin W-M1-hemiacetal (**3**) [9], rifamycin W (**4**) [17], protorifamycin I (**5**) [18], and saliniketal A (**8**) [19] by comparison of their spectroscopic data with those reported in the literature. 

**Figure 4:**
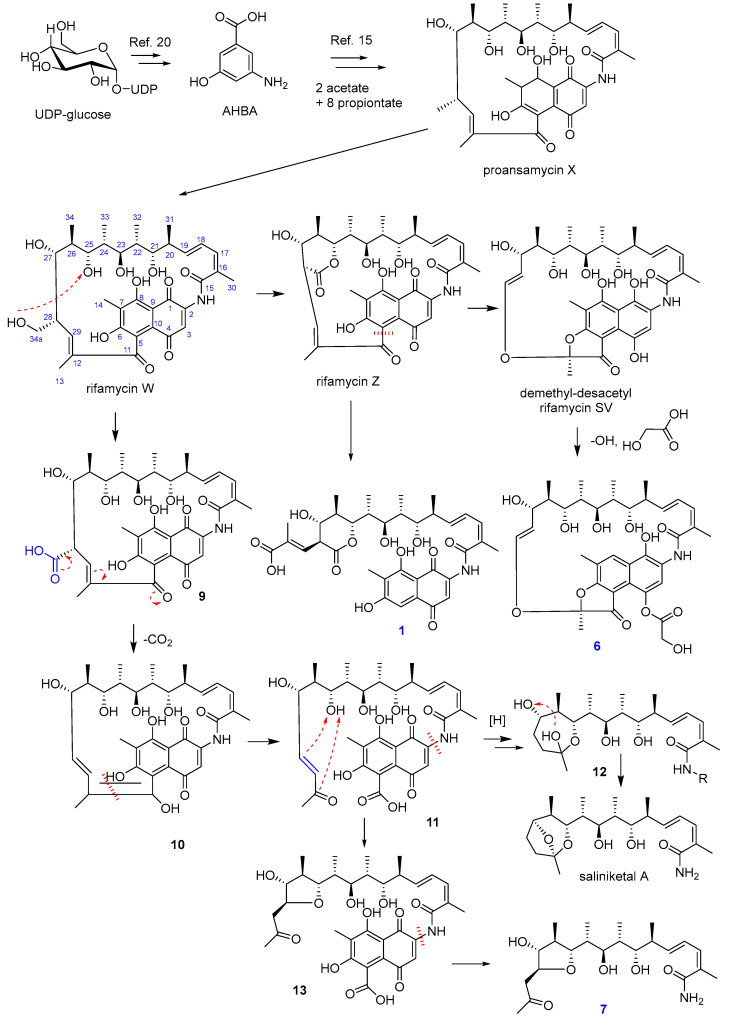
Plausible biosynthetic pathway of the new compounds.

Rifamycins are a group of antimicrobial drugs, and their mode of action, resistance, and biosynthesis have been well studied [20]. Previous studies showed that the biosynthesis of rifamycins starts with 3-amino-5-hydroxybenzoic acid (AHBA) and chain extension by various rifamycin biosynthetic genes. The ansa-chain was established from two acetates and eight propionates [15]. Numerous structures of naturally occurring rifamycins are mainly due to the extensive post-PKS enzymatic tailoring conducted by enzymes encoded by pathway-specific genes [15]. Similarly, the possible biosynthesis pathway of the new compounds was proposed, as shown in Figure 4. Firstly, proansamycin X was synthesized, and then the compound was converted to rifamycin W. Rifamycin W was oxidized to rifamycin Z, and then this compound was converted to demethly-desacetyl rifamycin SV. Compound **1** could be originated from rifamycin Z by oxidation and decyclization at the C-5 position. Compound **6** could be synthesized from demethly-desacetyl rifamycin SV by deoxidation at C-8 and adding glycolic acid (from an acetate unit) at the C-4 position. The biosynthesis of **7** was proposed to be similar to saliniketals. Rifamycin W was converted to a percussor (**11**) via several steps, and **11** could be reduced and cyclized to give a tetrahydrofuran-containing derivative (**13**), and then the napthoquinone moiety was eliminated to yield salinifuran (**7**).

Some isolated compounds were screened for their cytotoxicity against one normal, six solid, and seven blood cancer cell lines (Table 3). According to the guidelines of NCI, when a compound shows cytotoxicity with an IC_50_ value less than 4 µg/mL or 10 μM, the compound is considered to have cytotoxicity [21]. We defined the potency of compounds as significant (IC_50_ < 1 μM), moderate (IC_50_ = 1–10 μM), weak (IC_50_ = 10–30 μM), and non-cytotoxic (IC_50_ > 30 μM). Compound **1** showed moderate activity against all the tested cell lines with GI_50_ of 2.36–9.96 µM. Compound **6** exhibited weak cytotoxicity against solid cancer cells with GI_50_ values of 21.84–29.75 µM. Compound **7** showed weak activity against NALM6 (GI_50_ = 26.76 µM). Compound **8** was not active against all the tested cell lines (GI_50_ > 30 µM). From the activity results, it is noteworthy that rifamycin derivatives showed moderate or weak cytotoxicity, and the aromatic moiety is important for their cytotoxicity (**1** and **6**). Compounds **7** and **8** are composed of only an ansa-chain and showed weak or no cytotoxicity. The lack of a hydroxy group at the C-8 position (**6**) may reduce the activity. The results of this study were consistent with previous reports that naturally occurring rifamycins are generally not effective enough for new drug development. However, further research should be conducted to semi-synthesize their derivatives with lower toxicity against normal cells and better pharmacological properties. 

## 3. Materials and Methods

### 3.1. General Experimental Procedures

The 1D and 2D NMR spectra were measured by a Bruker 600 MHz spectrometer (Bruker BioSpin GmbH, Rheinstetten, Germany). UV–Vis spectra were obtained using a Shimadzu UV-1650PC spectrophotometer (Shimadzu Corporation, Kyoto, Japan). A JASCO FT/IR-4100 spectrophotometer (JASCO Corporation, Tokyo, Japan) was used to record IR spectra. A hybrid ion trap time-of-flight mass spectrometer (Shimadzu LC/MS-IT-TOF, Kyoto, Japan) was used for measuring high-resolution ESIMS experiments. HPLC was performed with a PrimeLine Binary pump (Analytical Scientific Instruments, Inc., El Sobrante, CA, USA) and a RI-101 detector (Shoko Scientific Co., Ltd., Yokohama, Japan). Semi-preparative HPLC was conducted using an ODS column (YMC-Pack-ODS-A, 250 × 10 mm i.d., 5 µM, Kyoto, Japan). A Rudolph Research Analytical Autopol III polarimeter (Rudolph Research Analytical, Hackettstown, NJ, USA) was used to record optical rotations. Mass culture was carried out using a 100 L fermenter (Fermentec Co., Ltd., Cheongju, Republic of Korea). All solvents were either HPLC grade or distilled prior to use. 

### 3.2. Isolation of the Microorganisms from Marine Sediment Samples

Marine sediment samples were obtained off the coast of Dokdo Island in the Republic of Korea during expeditions in May 2022. The sediment samples were obtained using a grab sampler at a depth of approximately 200 m below the water’s surface. The sediments were collected and stored at 5 °C upon return to the laboratory in sterile 50 mL conical tubes. *Actinomycetes* are spore-forming bacteria that can endure harsh environments and high temperatures. As a result, pretreatment with selective heating was employed to reduce non-spore-forming unwanted microorganisms. Each sample was weighed to 1.0 g, placed on a sterile plate, and kept at 60 °C for 30 min in a dry oven. The samples were then successively diluted to 10^−1^, 10^−2^, and 10^−3^ by sterile seawater, and each aliquot (100 µL) was spread on humic acid–vitamin agar (HV), actinomycetes isolation agar (AIA), and Bennett’s agar (BN) media. The plates were kept in a BOD (bio-oxygen demand) incubator at 28 °C for 1~4 weeks until colonies could be seen with the naked eye. Colonies that could be seen were picked up and transferred onto new BN agar plates. The purification procedure was carried out repeatedly several times until single pure colonies were obtained.

### 3.3. Isolation and Identification of the Strain 225DD-027

The strain 225DD-027 was isolated from humic acid–vitamin agar after being incubated for 12 days. It was identified as *Salinispora arenicola* according to its morphological characteristics and results of 16S-rRNA gene sequence analysis by Macrogen Inc. (Seoul, Republic of Korea). The sequence of 225DD-027 was submitted to GenBank under accession number OR084782. The strain is currently preserved in the Microbial Culture Collection, Korea Institute of Ocean Science and Technology (KIOST), with an accession number of 225DD-027, under the curatorship of Hee Jae Shin.

### 3.4. Fermentation of the Strain 225DD-027 and Extraction and Isolation of Metabolites

Bennett’s medium (BN, 1% glucose, 0.2% tryptone, 0.1% yeast extract, 0.1% beef extract, 0.5% glycerol, sea salts 32 g/L, and agar 17 g/L for agar medium) was used to conduct the seed and mass cultures of the strain 225DD-027. Firstly, the strain was cultured on Bennett’s agar medium in a Petri dish for 14 days. The actively grown colonies were transferred aseptically into a 100 mL conical flask containing 50 mL of Bennett’s broth medium and incubated on a rotary shaker (140 rpm) at 28 °C for 9 days. An aliquot (10 mL) was aseptically inoculated into a 2.0 L flask containing 1.0 L of BN broth. The strain was incubated at 28 °C for 7 days on a rotary shaker at 140 rpm, and then the culture broth was inoculated into a 100 L fermenter filled with 70 L of BN broth. The mass culture was carried out at 28 °C for 16 days and then harvested. The culture was separated into supernatant and mycelium by continuous centrifugation at 60,000 rpm, and the supernatant was extracted twice with an equal volume of EtOAc (70 L × 2). The EtOAc layer was evaporated under reduced pressure to obtain a crude extract (8.0 g). The extract was then fractionated into 15 fractions (fractions 1 to 15) by vacuum liquid chromatography on an ODS column using a stepwise elution with 3 × 300 mL each of 20%, 40%, 60%, and 80% MeOH in H_2_O and 100% MeOH. The F7 fraction was subjected to a semi-preparative HPLC (YMC-Pack ODS-A, 250 × 10 mm i.d., 5 μm, flow rate 2.0 mL/min) with an isocratic elution of 45% MeOH in H_2_O to obtain compound **7** (2.0 mg, *t_R_* = 42 min). The F9 fraction was purified using semi-preparative HPLC (YMC-Pack ODS-A, 250 × 10 mm i.d., 5 μm, flow rate 2.0 mL/min) with an isocratic elution of 30% MeCN in H_2_O to yield compounds **4** (3.0 mg, *t_R_* = 40 min) and **8** (3.5 mg, *t_R_* = 52 min). Compounds **5** (5.0 mg, *t_R_* = 65 min) and **6** (3.3 mg, *t_R_* = 75 min) were isolated from the F10 fraction using a semi-preparative HPLC (YMC-Pack ODS-A, 250 × 10 mm i.d., 5 μm, flow rate 2.0 mL/min) with an isocratic elution of 30% MeCN in H_2_O. The F11 fraction was purified using a semi-preparative HPLC (YMC-Pack ODS-A, 250 × 10 mm i.d., 5 μm, flow rate 2.0 mL/min) with an isocratic elution of 54% MeCN in H_2_O to yield compounds **1** (1.2 mg, *t_R_* = 32 min), **2** (1.5 mg, *t_R_* = 26 min), and **3** (1.5 mg, *t_R_* = 22 min).

Salinisporamycin C (**1**): orange powder, ${\left[\alpha \right]}_{D}^{20}$ − 30.5 (*c* 0.1, MeOH); UV (MeOH) *λ*_max_ (log *ε*) 218 (4.65), 273 (4.30) nm; IR *ν*_max_ 3364, 2976, 2880, 1632, 1600, 1496, 1374, 1062, 1021 cm^−1^; HRESIMS *m*/*z* 670.2852, calculated for [C_35_H_44_NO_12_]^+^, 670.2858; for ^1^H NMR (CD_3_OD, 600 MHz), see Table 1; for ^13^C NMR (CD_3_OD, 150 MHz), see Table 1.

Salinisporamycin D (**6**): orange powder, ${\left[\alpha \right]}_{D}^{20}$ + 35.0 (*c* 0.1, MeOH); UV (MeOH) *λ*_max_ (log *ε*) 222 (4.50), nm; IR *ν*_max_ 3364, 2966, 2851, 1759, 1643, 1590, 1222, 1099 cm^−1^; HRESIMS *m*/*z* 706.2809, calculated for [C_36_H_45_NO_12_Na]^+^, 706.2834; for ^1^H NMR (CD_3_OD, 600 MHz), see Table 1; for ^13^C NMR (CD_3_OD, 150 MHz), see Table 1.

Salinifuran A (**7**): colorless solid, ${\left[\alpha \right]}_{D}^{20}$ − 50.0 (*c* 0.1, MeOH); UV (MeOH) *λ*_max_ (log *ε*) 204 (5.10), nm; IR *ν*_max_ 3339, 2961, 2922, 1706, 1660, 1596, 1458, 1024 cm^−1^; HRESIMS *m/z* 434.2496, calculated for [C_22_H_37_NO_6_Na]^+^, 434.2513; for ^1^H NMR (CD_3_OD, 600 MHz), see Table 2; for ^13^C NMR (CD_3_OD, 150 MHz), see Table 2.

### 3.5. Cytotoxicity Assay

The cytotoxic activities of **1** and **6**–**8** against adherent cells and suspension cells were conducted by SRB (sulforhodamine B) assay [22] and CellTiter-Glo assay [23], respectively, as described earlier. The compounds were dissolved and serially diluted in dimethyl sulfoxide. GraphPad Prism 8 (GraphPad Software Inc., San Diego, CA, USA) was used to determine GI_50_ values. Cancer and normal cell lines were purchased from the Japanese Cancer Research Resources Bank (JCRB) (NUGC-3, JCRB Cell Bank/Cat. # JCRB0822), the DSMZ-German Collection of Microorganisms and Cell Cultures (RPMI-8402, DSMZ/Cat # ACC 290; WSU-DLCL2, DSMZ/Cat # ACC 575), and the American Type Culture Collection (ATCC) (PC-3, ATCC/Cat. # CRL-1435; MDA-MB-231, ATCC/Cat. # HTB-26; ACHN, ATCC/Cat. # CRL-1611; NCI-H23, ATCC/Cat. # CRL-5800; HCT-15, ATCC/Cat. # CCL-225; HL-60, ATCC/Cat. # CCL-240; Raji, ATCC/Cat # CCL-86; K562, ATCC/Cat # CCL-243; NALM6, ATCC/Cat # CRL-3273; U266, ATCC/Cat # TIB-196); RPMI-1788, ATCC/Cat # TIB-156). Cells were cultured in Dulbecco’s Modified Eagles medium (ACHN, MDA-MB-231), RPMI-1640 medium (HCT-15, PC-3, NUGC-3, NCI-H23, Raji, NALM6, U266, WSU-DLCL2, RPMI-1788) or Improved Minimum Essential Medium (K562, HL-60), supplemented with fetal bovine serum and penicillin-streptomycin. 

## 4. Conclusions

In conclusion, we have isolated eight rifamycin-related polyketides from the marine-derived actinomycetal strain *Salinispora arenicola* 225DD-027, including three new compounds (**1**, **6**, and **7**). The gross structures of the new compounds were elucidated by detailed analysis of their spectroscopic data (HRESIMS, 1D, and 2D NMR). Their absolute configuration was established by consideration of their biosynthetic relationship and comparison of their NMR chemical shifts, ^1^H-^1^H coupling constants, and ECD spectra with those of the co-isolated derivatives. The new compounds were evaluated for their cytotoxicity against one normal, six solid, and seven blood cancer cell lines, and **1** displayed moderate cytotoxicity against all the tested cancer cell lines with GI_50_ ranging from 2.36 to 9.96 µM. Further investigations should be performed to comprehend the mechanism of action of the active metabolites. The findings of this report have enriched the biological and chemical diversities of naturally occurring polyketides from the genus *Salinispora*.

## Figures and Tables

**Table 1:** ^1^H and ^13^C NMR data for **1** and **6**.

	1		6		
No	*δ*_H_, Mult (J in Hz)	*δ*_C_	No	*δ*_H_, Mult (J in Hz)	*δ*_C_
1		183.8	1		144.1
2		142.4	2		125.0
3	7.63, s	117.1	3	7.21, s	119.4
4		186.5	4		139.7
5	7.05, s	108.7	5		121.5
6		163.8	6		175.5
7		118.1	7		125.0
8		165.1	8	8.61, s	137.2
9		108.2	9		122.6
10		132.2	10		112.2
11		171.4	11		197.6
12		134.8	12		108.5
13	1.91, d, (1.3)	13.5	13	1.68, s	21.5
14	2.11, s	8.0	14	2.49, s	15.1
15		169.9	15		172.3
16		129.4	16		132.0
17	6.48, d, (11.1)	138.7	17	6.30, d, (10.7)	134.1
18	6.82, dd, (15.0, 11.2)	127.7	18	6.43, dd, (15.4, 11.2)	124.9
19	6.03, dd, (15.1, 8.0)	145.7	19	5.98, dd, (15.5, 5.7)	141.7
20	2.44. m	42.2	20	2.35, m	39.8
21	3.76, d, (9.8)	75.9	21	3.95, d, (9.4)	73.4
22	1.94, m	35.0	22	1.79, m	34.5
23	3.65, dd, (10.1, 2.6)	77.7	23	3.43, d, (10.3)	78.3
24	2.07. m	38.7	24	1.35, m	39.4
25	4.60, d, (11.0)	83.1	25	3.62, d, (10.6)	71.8
26	1.93, m	39.1	26	1.06, m	41.8
27	3.55, t, (9.6)	74.5	27	4.38, d, (5.6)	68.5
28	3.45, t, (9.6)	51.6	28	5.22, dd, (12.4, 6.0)	125.0
29	6.68, dd, (9.6, 1.2)	137.1	29	6.19, d, (12.5)	142.1
30	2.09, s	20.5	30	2.07, s	20.5
31	1.01, d, (6.8)	16.9	31	0.92, d, (6.9)	18.0
32	1.10, d, (7.0)	11.6	32	1.03, d, (6.9)	11.3
33	0.89, d, (6.9)	9.3	33	0.60, d, (6.8)	8.9
34	1.08, d, (6.5)	13.1	34	−0.64, d, (6.7)	8.8
34a		173.0	35		174.0
			36	4.70, d, (16.9) 4.56, d, (16.9)	62.1

**Table 2:** ^1^H and ^13^C NMR data for **7**.

No	*δ*_H_, Mult (J in Hz)	*δ*_C_	No	*δ*_H_, Mult (J in Hz)	*δ*_C_
1		175.1	13	3.46, t (8.4)	82.7
2		131.4	14a	3.95, td (8.7, 3.5)	80.8
3	6.17, br d (11.1)	134.1	14b		
4	6.58, dd (15.2, 11.1)	128.3	15a	2.75 dd (15.6, 3.5)	48.5
5	5.78, dd (15.2, 8.3)	142.1	15b	2.63 dd (15.6, 8.9)	
6	2.34, m	42.3	16		210.3
7	3.73, dd (9.3, 1.3)	75.6	17	2.18, s	30.6
8	1.89, m	36.3	18	1.95, s	20.9
9	3.50, dd (7.9, 4.6)	78.9	19	0.95, d (6.9)	17.1
10	1.81, m	38.5	20	0.98, d (7.0)	10.9
11	4.05, dd (10.2, 1.2)	83.5	21	0.95, d (6.9)	10.6
12	2.00, m	44.1	22	1.05, d (6.5)	14.2

**Table 3:** Growth inhibition (GI_50_, µM) of **1** and **6**-**8** against cancer cell lines.

	1	6	7	8	Adr.
ACHN	8.45	>30	>30	>30	0.168
MDA-MB-231	8.79	>30	>30	>30	0.159
HCT-15	9.25	24.20	>30	>30	0.137
PC-3	8.46	26.59	>30	>30	0.144
NUGC-3	7.41	29.75	>30	>30	0.136
NCI-H23	8.05	21.84	>30	>30	0.151
HL-60	9.96	4.79	>30	>30	0.021
Raji	5.37	12.05	>30	>30	0.003
K562	7.17	9.53	>30	>30	0.057
RPMI-8402	4.75	2.45	>30	>30	0.014
NALM6	4.28	4.76	26.76	>30	0.002
U266	2.63	2.30	>30	>30	0.034
WSU-DLCL2	2.36	5.40	>30	>30	0.002
RPMI-1788	8.47	4.93	nt	nt	0.014

Adr.: Adriamycin as a positive control. nt: not tested. Six solid [ACHN (renal), MDA-MB-231 (breast), HCT-15 (colon), PC-3 (prostate), NUGC-3 (stomach), and NCI-H23 (lung)], seven blood [(HL-60 (acute myelogenous leukemia, AML), Raji (Burkitt’s lymphoma), K562 (chronic myelogenous leukemia, CML), RPMI-8402 (T cell acute lymphocytic leukemia, T-ALL), NALM6 (B cell acute lymphocytic leukemia, B-ALL), U266 (multiple myeloma), WSU-DLCL2 (diffuse large B cell lymphoma, DLBCL) cells lines and a normal [RPMI-1788 (B lymphocytes)] cell line.

## Data Availability

The data presented in the article are available in the Supplementary Materials.

## Supplementary Materials

The following supporting information can be downloaded at https://www.mdpi.com/article/10.3390/md21090494/s1. Figure S1. Structures of some analogs of the isolated compounds. Figure S2. ^1^H NMR spectrum of **1**. Figure S3. ^13^C NMR spectrum of **1**. Figure S4. HSQC spectrum of **1**. Figure S5. ^1^H-^1^H COSY spectrum of **1**. Figure S6. HMBC spectrum of **1**. Figure S7. Selective 1D NOESY spectrum of **1** (irradiated at H-17). Figure S8. Selective 1D NOESY spectrum of **1** (irradiated at H-27). Figure S9. Selective 1D NOESY spectrum of **1** (irradiated at H-28). Figure S10. Selective 1D NOESY spectrum of **1** (irradiated at H-25). Figure S11. HRESIMS data of **1**. Figure S12. ^1^H NMR spectrum of **6**. Figure S13. ^13^C NMR spectrum of **6**. Figure S14. HSQC spectrum of **6**. Figure S15. ^1^H-^1^H COSY spectrum of **6**. Figure S16. HMBC spectrum of **6**. Figure S17. NOESY spectrum of **6**. Figure S18. HRESIMS data of **6**. Figure S19. ^1^H NMR spectrum of **7**. Figure S20. ^13^C NMR spectrum of **7**. Figure S21. HSQC spectrum of **7**. Figure S22. ^1^H-^1^H COSY spectrum of **7**. Figure S23. HMBC spectrum of **7**. Figure S24. Band selective HMBC of **7**. Figure S25. Selective 1D NOESY spectrum of **7** (irradiated at H-3). Figure S26. Selective 1D NOESY spectrum of **7** (irradiated at H-11). Figure S27. Selective 1D NOESY spectrum of **7** (irradiated at H-13). Figure S28. Selective 1D NOESY spectrum of **7** (irradiated at H-14). Figure S29. HRESIMS data of **7**. Figure S30. Comparison of ECD spectra of **7** and saliniketal A (**8**). Figure S31. Results of cytotoxicity test and dose–response curves for compounds **1** and **6**–**8**. Table S1. Comparison of chemical shifts of **1** and **2**. Table S2. Comparison of chemical shifts of **7** and **8**.
