# Supplementary material for: Rifamycin-Related Polyketides from a Marine-Derived Bacterium Salinispora arenicola and Their Cytotoxic Activity
Source: Mar Drugs. 2023 Sep 15;21(9):494. doi: 10.3390/md21090494 (PMC10532523; doi:10.3390/md21090494)
Supplement: Supplementary file 1 [file marinedrugs-21-00494-s001.zip › marinedrugs-2579795-supplementary.pdf]

Rifamycin-Related Polyketides from a Marine-Derived Bacterium *Salinispora arenicola* and Their Cytotoxic Activity

Cao Van Anh [1,†], Jong Soon Kang [2], Jeong-Wook Yang [2], Joo-Hee Kwon [2], Chang-Su Heo [1,3], Hwa-Sun Lee [1], and Hee Jae Shin [1,3,*]

Contents

Figure S 1. Structures of some analogs of the isolated compounds. 4
Figure S 2. ^{1}H NMR spectrum of **1**. 8
Figure S 3. ^{13}C NMR spectrum of **1**. 9
Figure S 4. HSQC spectrum of **1**. 10
Figure S 5. ^{1}H-^{1}H COSY spectrum of **1**. 11
Figure S 6. HMBC spectrum of **1**. 12
Figure S 7. Selective 1D NOESY spectrum of **1** (irradiated at H-17). 13
Figure S 8. Selective 1D NOESY spectrum of **1** (irradiated at H-27). 15
Figure S 9. Selective 1D NOESY spectrum of **1** (irradiated at H-28). 16
Figure S 10. Selective 1D NOESY spectrum of **1** (irradiated at H-25). 17
Figure S 11. HRESIMS data of **1**. 18
Figure S 12. ^{1}H NMR spectrum of **6**. 19
Figure S 13. ^{13}C NMR spectrum of **6**. 20
Figure S 14. HSQC spectrum of **6**. 21
Figure S 15. ^{1}H-^{1}H COSY spectrum of **6**. 22
Figure S 16. HMBC spectrum of **6**. 23
Figure S 17. NOESY spectrum of **6**. 24
Figure S 18. HRESIMS data of **6**. 25
Figure S 19. ^{1}H NMR spectrum of **7**. 29
Figure S 20. ^{13}C NMR spectrum of **7**. 30
Figure S 21. HSQC spectrum of **7**. 31
Figure S 22. ^{1}H-^{1}H COSY spectrum of **7**. 32
Figure S 23. HMBC spectrum of **7**. 33
Figure S 24. Band selective HMBC of **7**. 34
Figure S 25. Selective 1D NOESY spectrum of **7** (irradiated at H-3). 35
Figure S 26. Selective 1D NOESY spectrum of **7** (irradiated at H-11). 36
Figure S 27. Selective 1D NOESY spectrum of **7** (irradiated at H-13). 37
Figure S 28. Selective 1D NOESY spectrum of **7** (irradiated at H-14). 38
Figure S 29. HRESIMS data of **7**. 39
Figure S 30. Comparison of ECD spectra of **7** and saliniketal A (**8**). 40
Figure S 31. Results of cytotoxicity test and dose-response curves for compounds **1** and **6-8.** 45

Table S 1. Comparison of chemical shifts of **1** and **2**. 5
Table S 2. Comparison of chemical shifts of **7** and **8**. 26

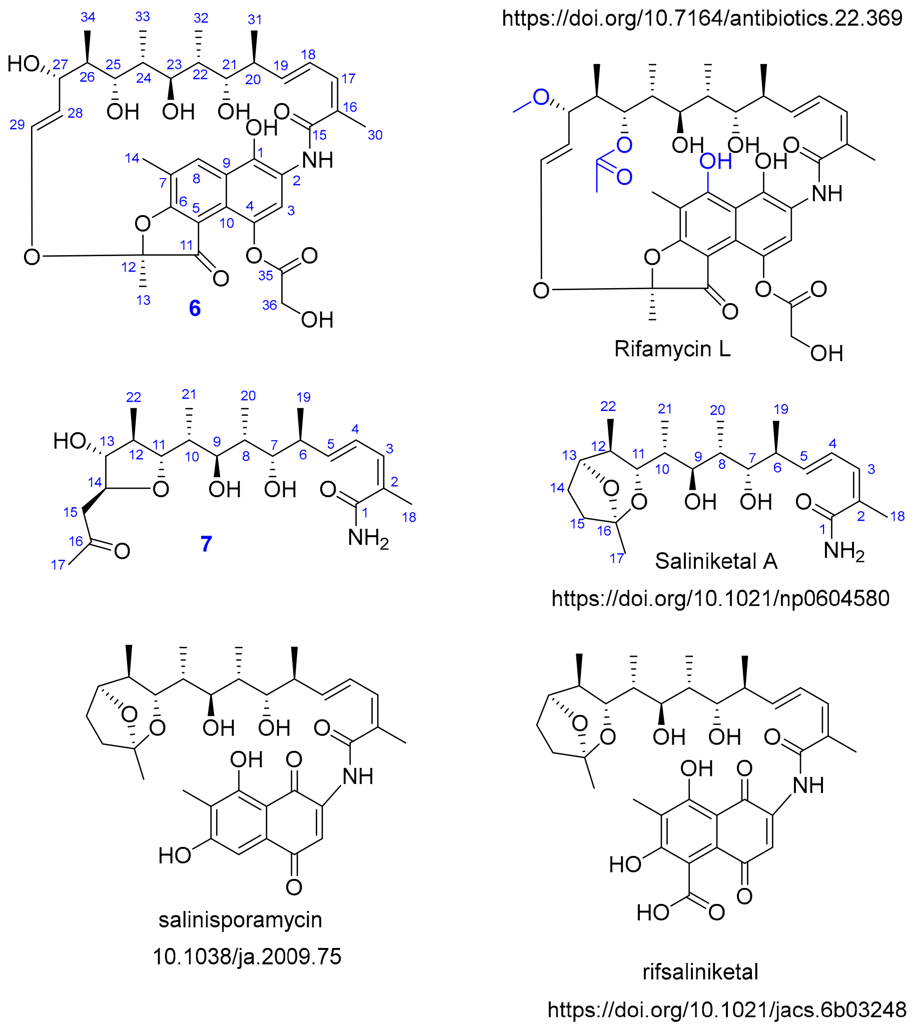

Figure S1. Structures of some analogs of the isolated compounds.

Table S1. Comparison of chemical shifts of **1** and **2**.

	**1**		**2**	
	δ_H	δ_C	δ_H	δ_C
1		183.8		184.1
2		142.4		142.4
3	7.63, s	117.1	7.61, s	117.2
4		186.5		186.5
5	7.05, s	108.7	7.02, s	108.8
6		163.8		164.0
7		118.1		118.3
8		165.1		165.2
9		108.2		131.1
10		132.2		132.4
11		171.4		172.3
12		134.8		131.1
13	1.91, d, *J* =1.3	13.5	1.88, s	13.3

14	2.11, s	8.0	2.09, s	8.2
15		169.9		170.0
16		129.4		129.4
17	6.48, d, J = 11.1	138.7	6.50, d, J = 10.8	139.1
18	6.82, dd, J = 15.0, 11.2	127.7	6.86, dd, J = 14.9, 12.4	127.8
19	6.03, dd, J = 15.1, 8.0	145.7	6.07, m	146.4
20	2.44. m	42.2	2.47, m	42.5
21	3.76, d, J =9.8	75.9	3.83, m	75.8
22	1.94, m	35.0	1.96, m	36.5
23	3.65, dd, J = 10.1, 2.6	77.7	3.62, m	78.5
24	2.07. m	38.7	2.02, m	37.0
25	4.60, d, J = 11.0	83.1	4.21, m	72.7
26	1.93, m	39.1	1.61, m	41.3
27	3.55, t, J = 9.6	74.5	3.56, m	73.4
28	3.45, t, J = 9.6	51.6	2.61, m	50.7
29	6.68, dd, J = 9.6, 1.2	137.1	6.78, d, J = 10.1	142.4
30	2.09, s	20.5	2.08, s	20.7

31	1.01, d, *J* =6.8	16.9	1.02, s	17.2
32	1.10, d, *J* = 7.0	11.6	1.00, s	11.2
33	0.89, d, *J* =6.9	9.3	0.98, s	10.9
34	1.08, d, *J* = 6.5	13.1	0.96, s	12.8
34a		173.0	5.08, d, *J* = 3.3	94.5
Biomolecules **2021**, *11*(7), 920; https://doi.org/10.3390/biom11070920				

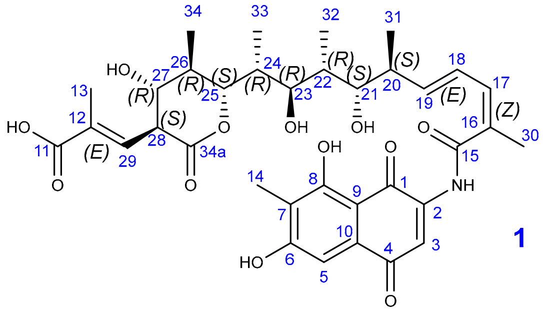

1

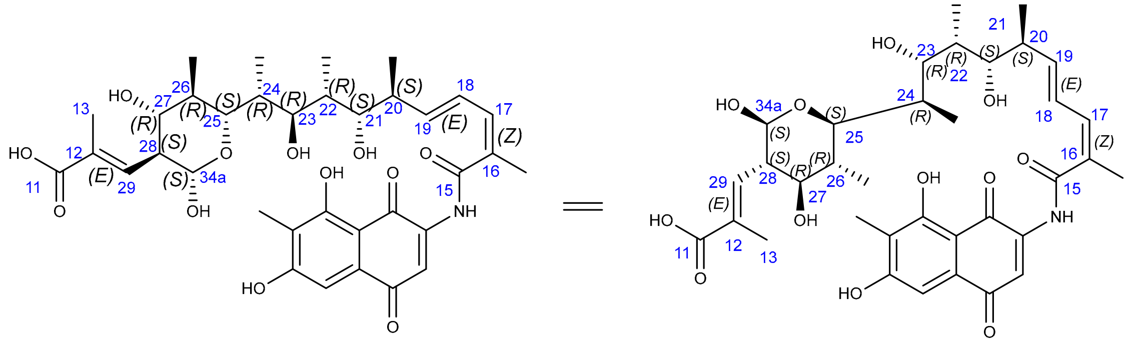

2 drawn in different ways

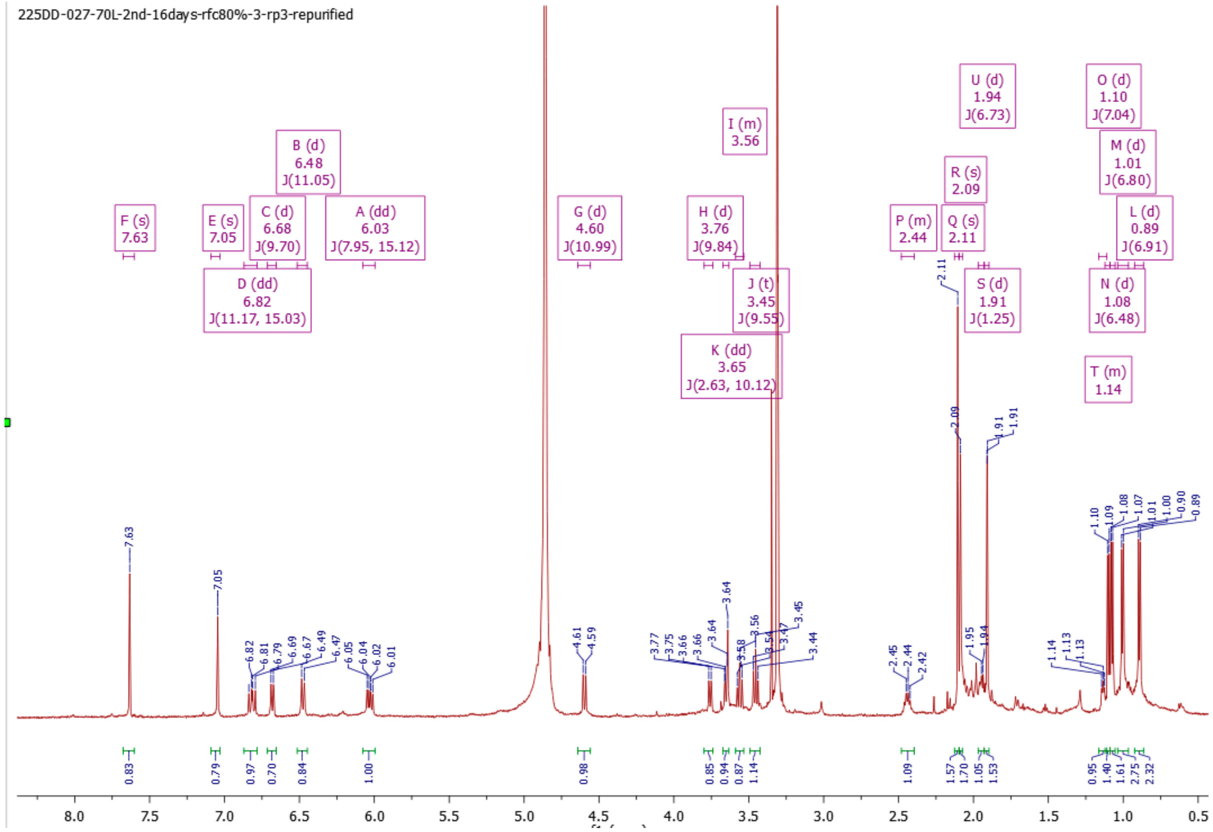

Figure S2. ^{1}H NMR spectrum of **1**.

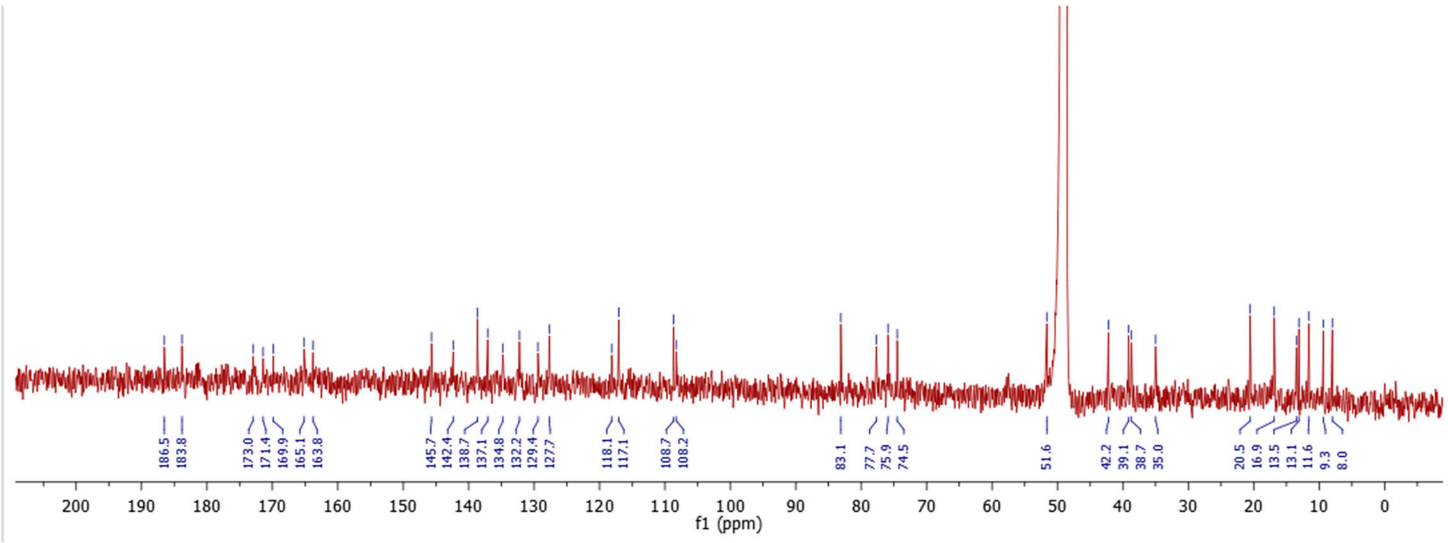

Figure S3. ^{13}C NMR spectrum of **1**.

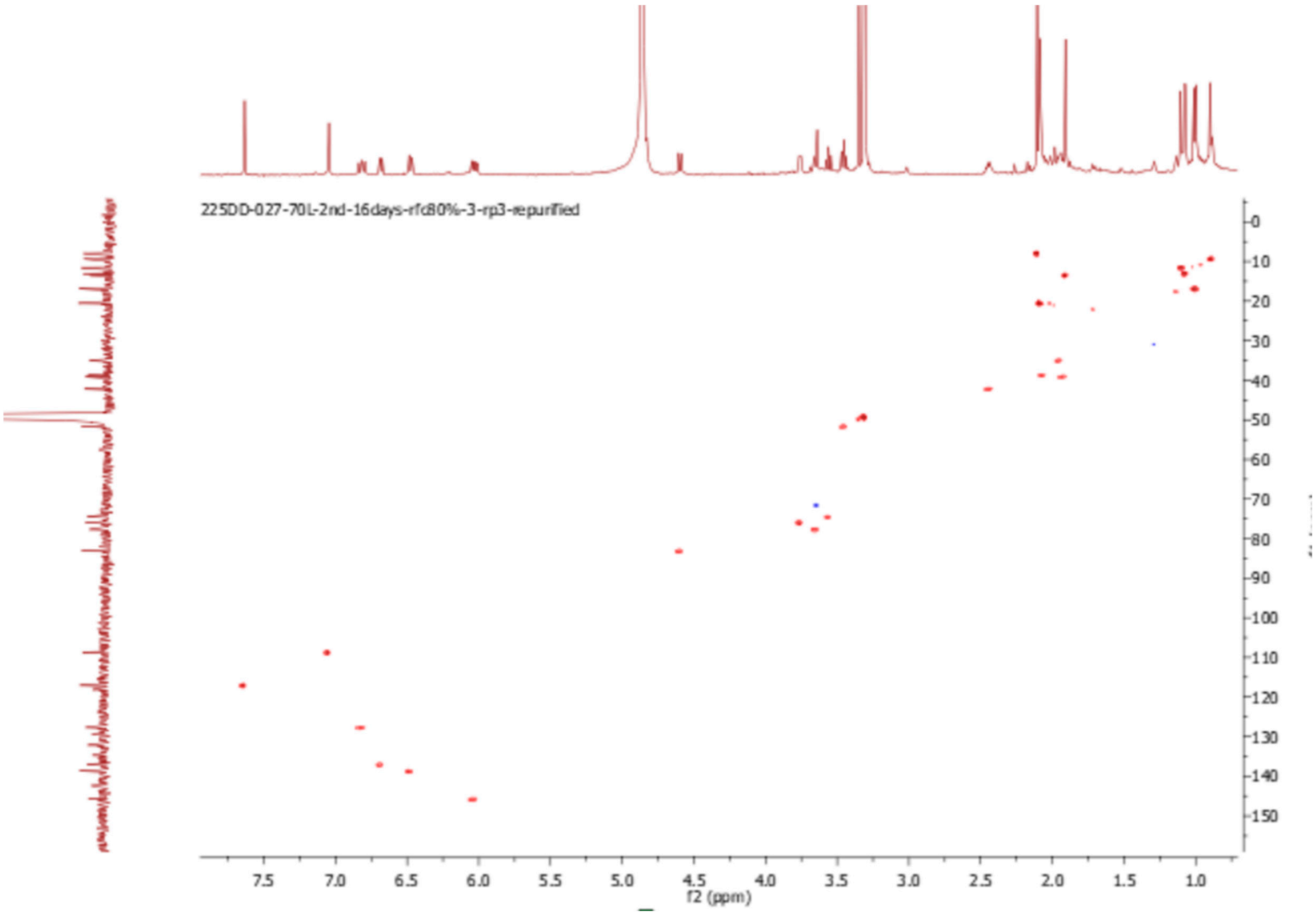

Figure S4. HSQC spectrum of **1**.

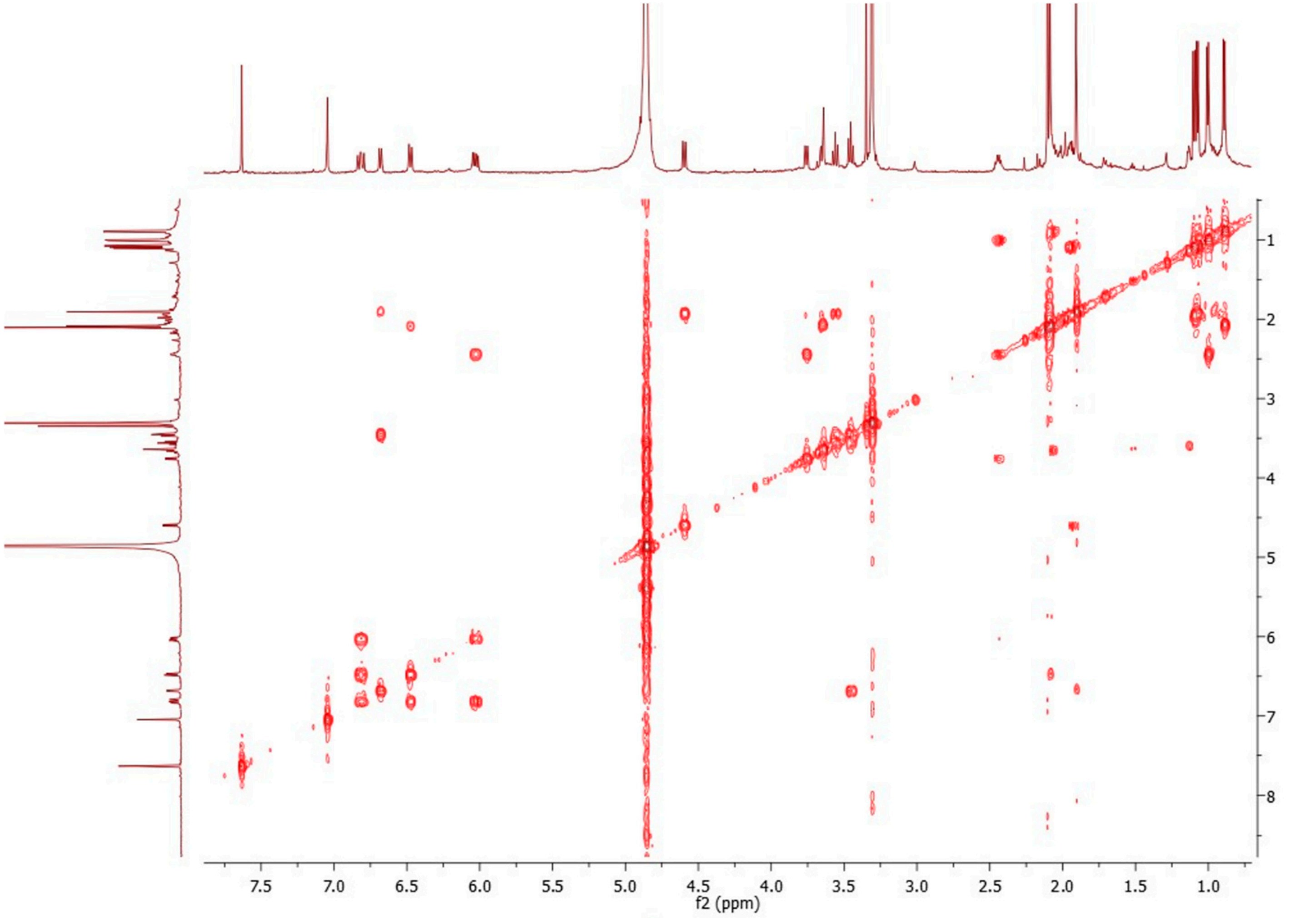

Figure S5. ^{1}H-^{1}H COSY spectrum of **1**.

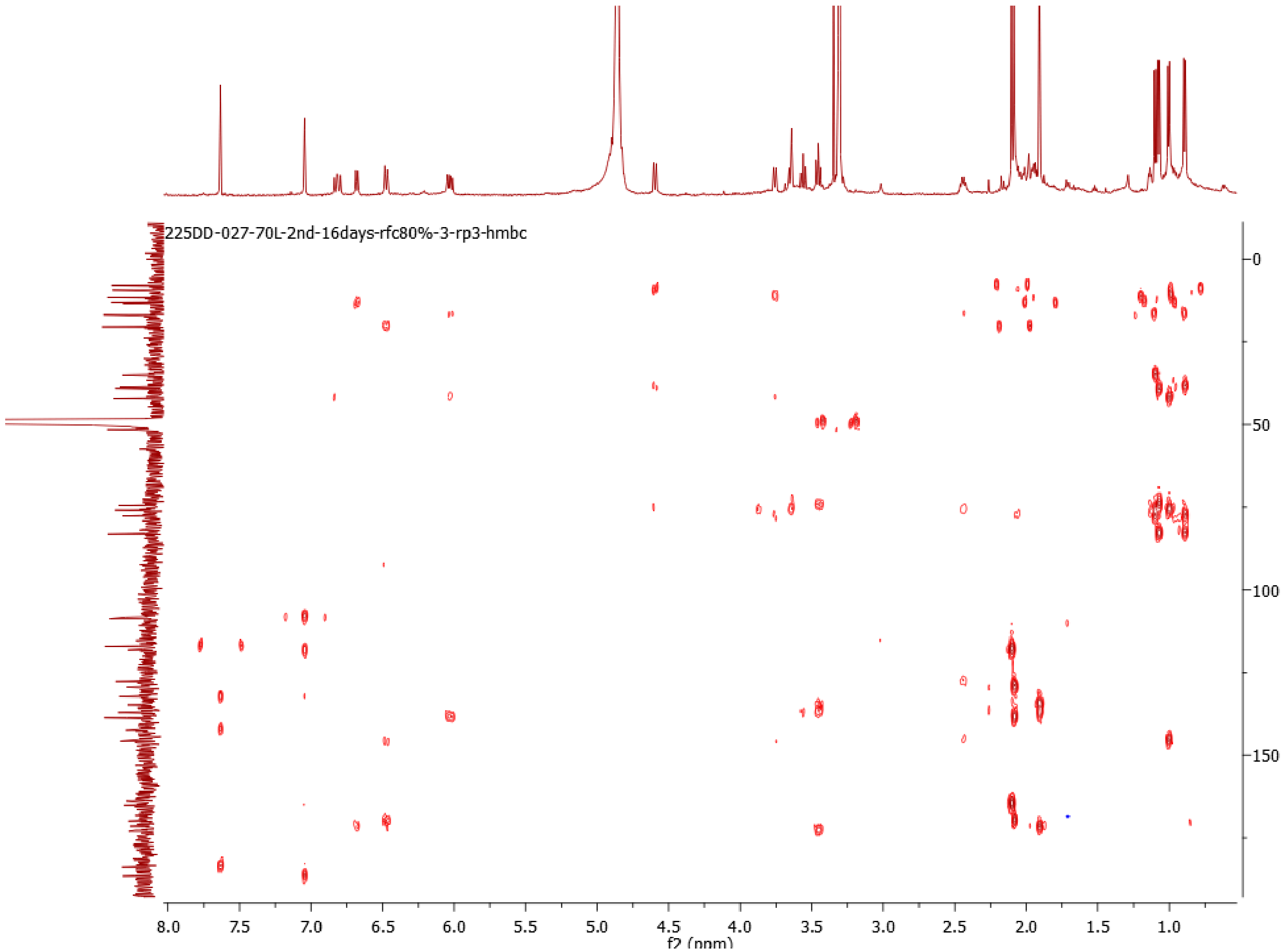

Figure S6. HMBC spectrum of **1**.

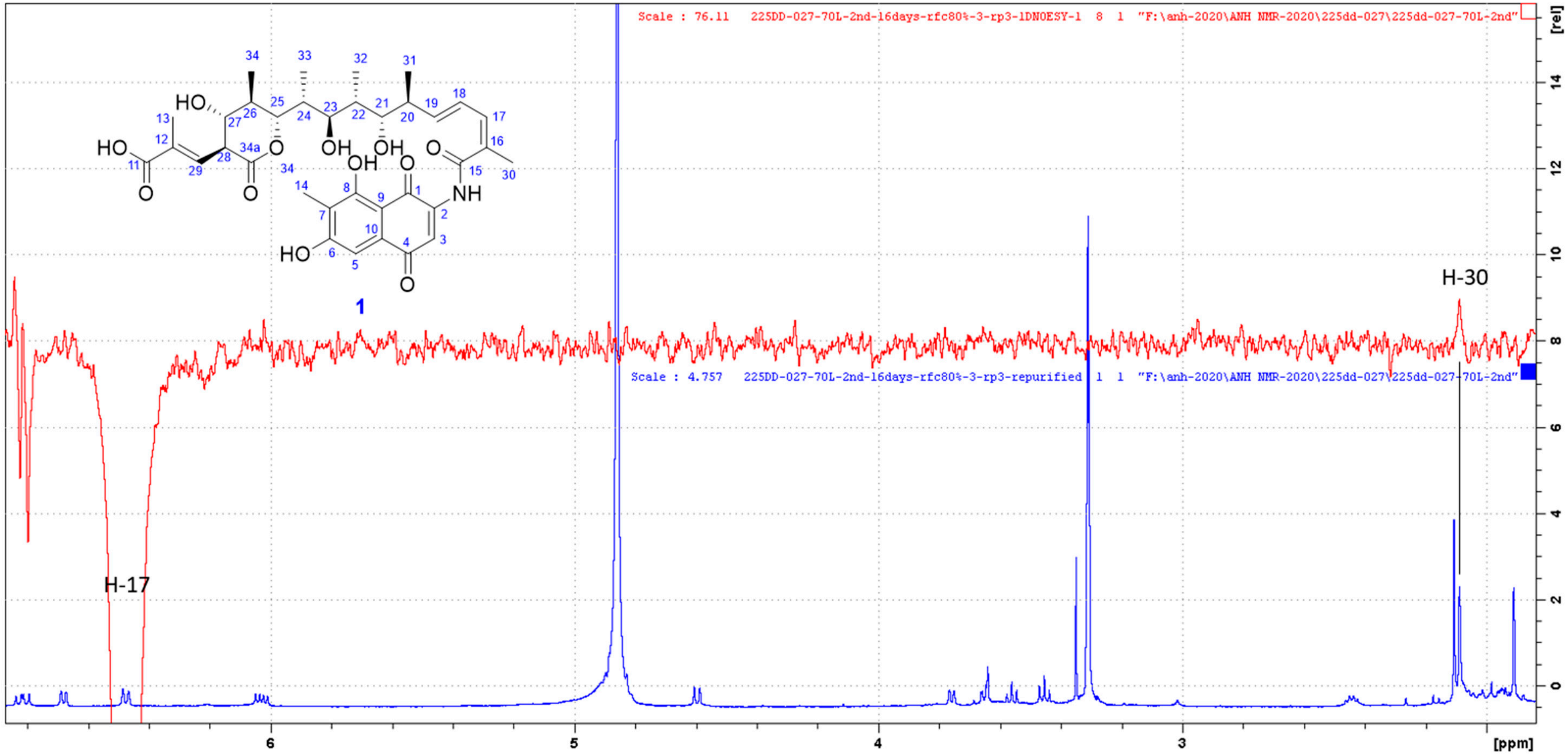

Figure S7. Selective 1D NOESY spectrum of **1** (irradiated at H-17).

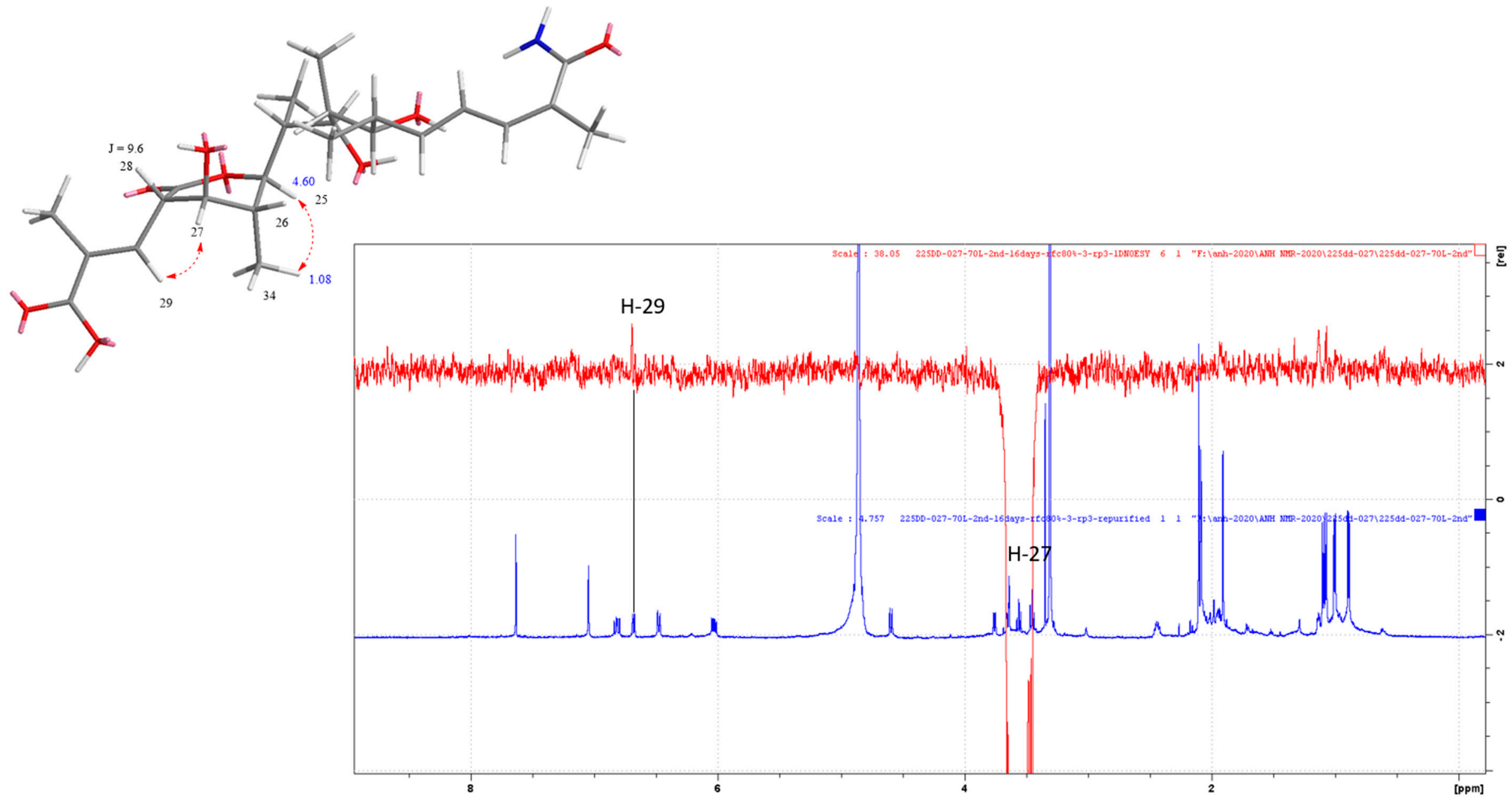
J = 9.6
28
4.60
25
27
26
29
34
1.08
H-29
H-27
[rel]
2
0
-2
8
6
4
2
[ppm]

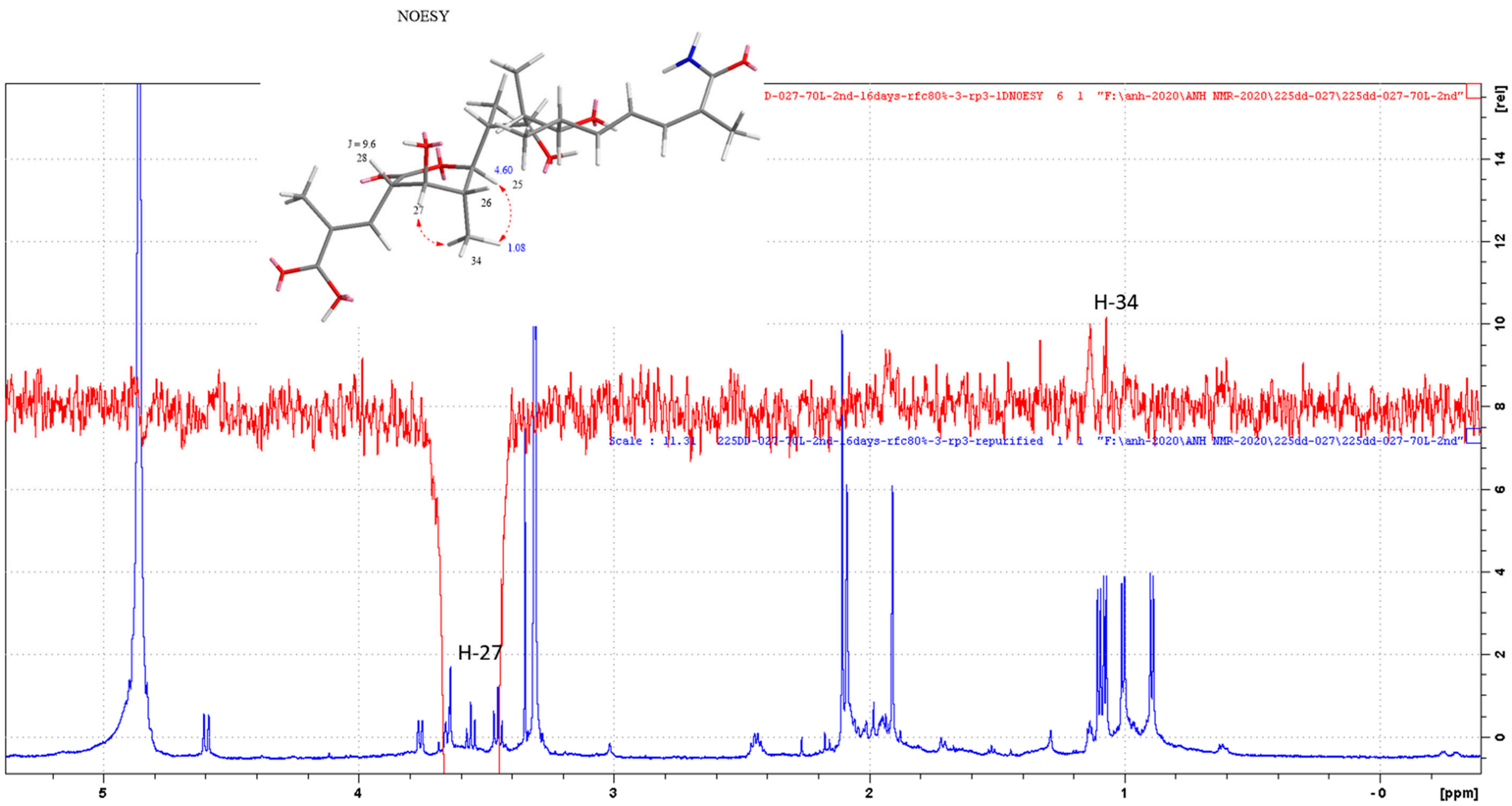

Figure S8. Selective 1D NOESY spectrum of **1** (irradiated at H-27).

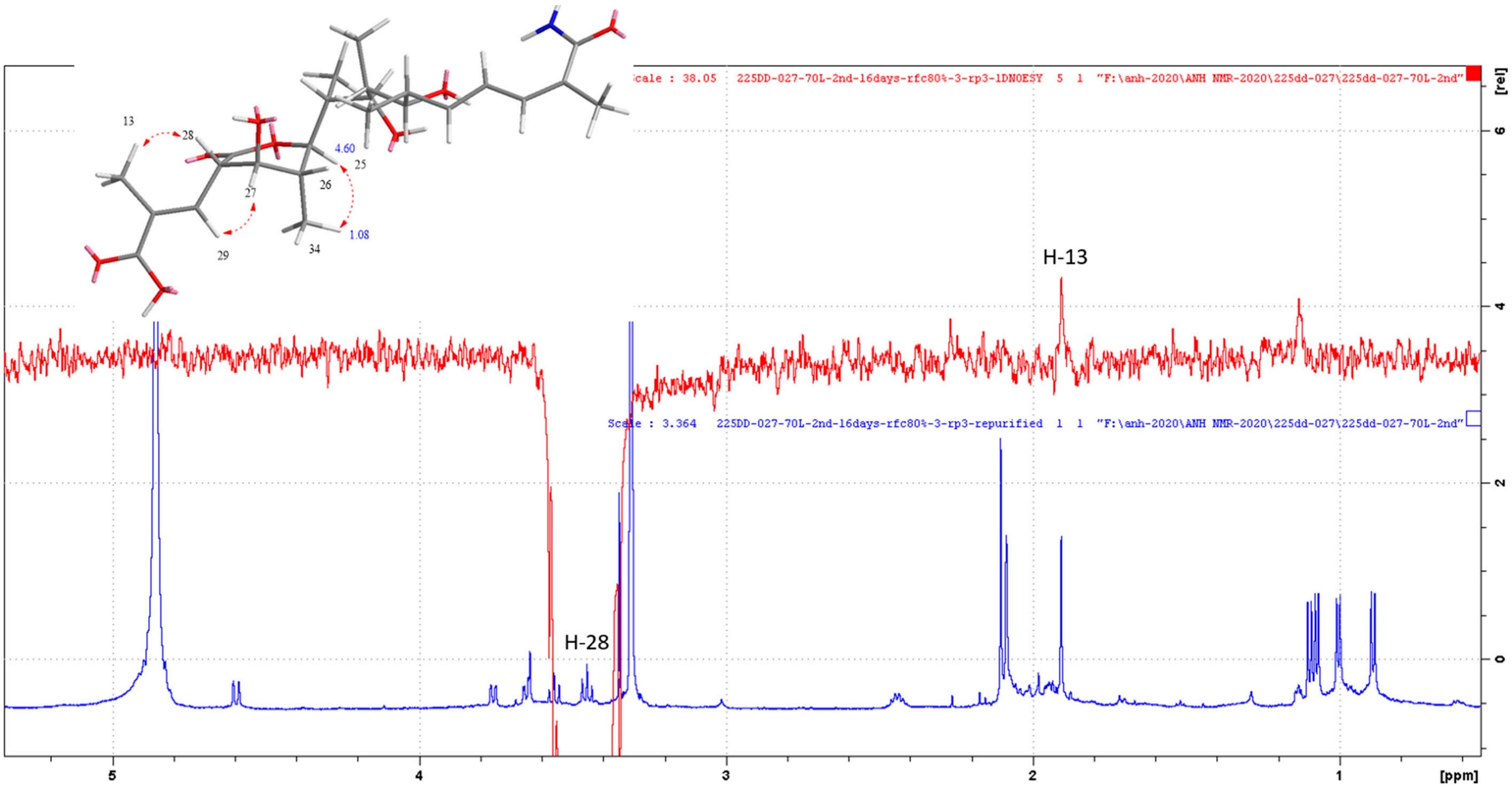

Figure S9. Selective 1D NOESY spectrum of **1** (irradiated at H-28).

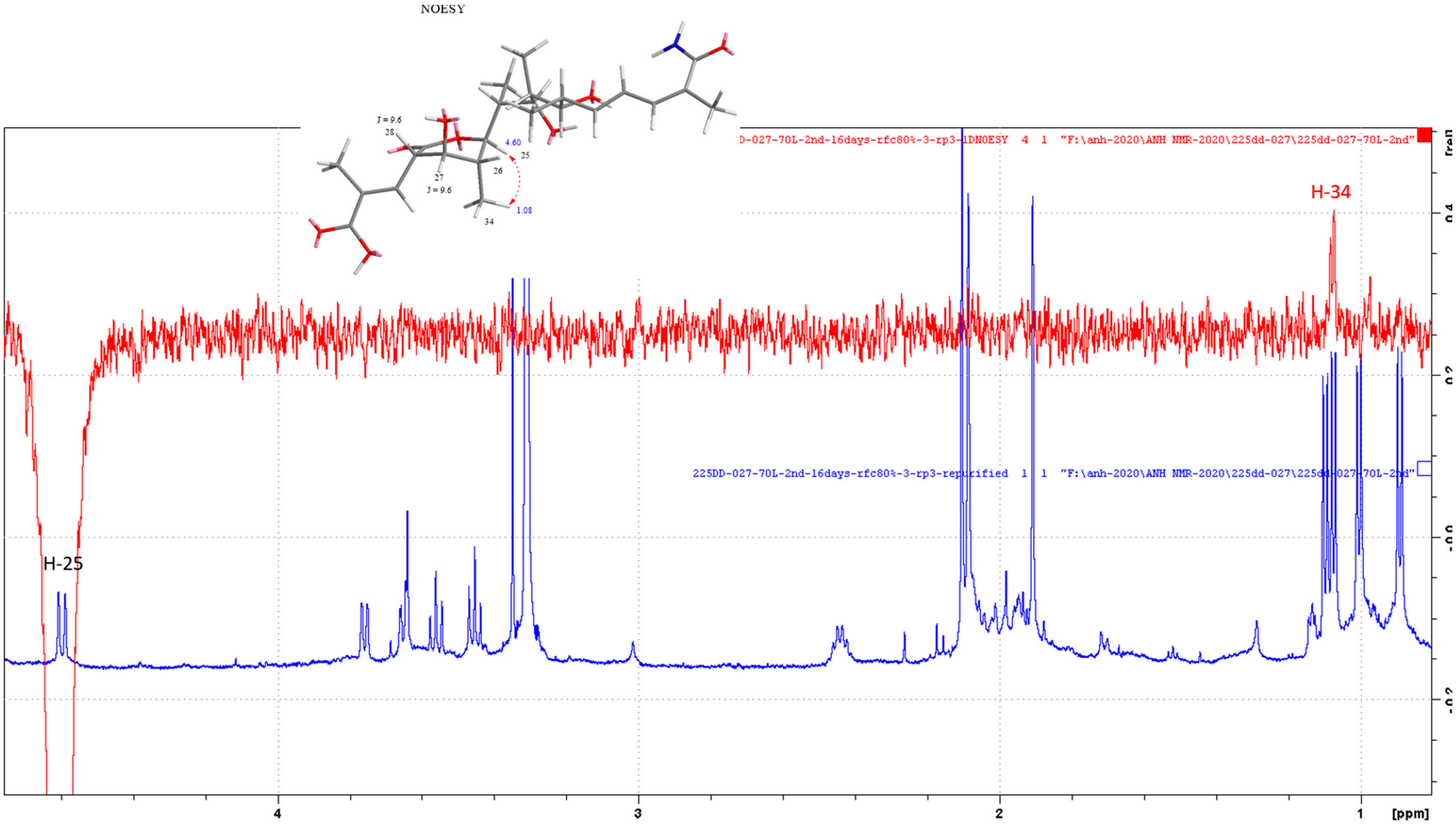

Figure S10. Selective 1D NOESY spectrum of **1** (irradiated at H-25).

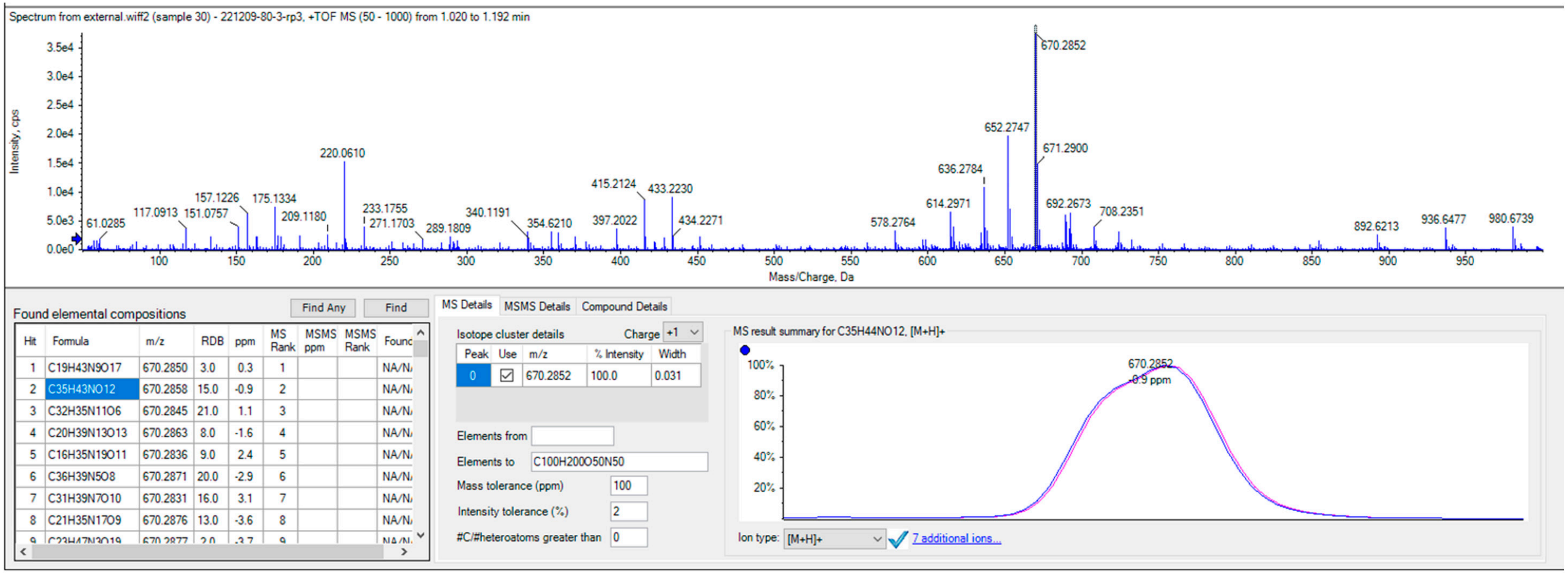

Figure S11. HRESIMS data of **1**.

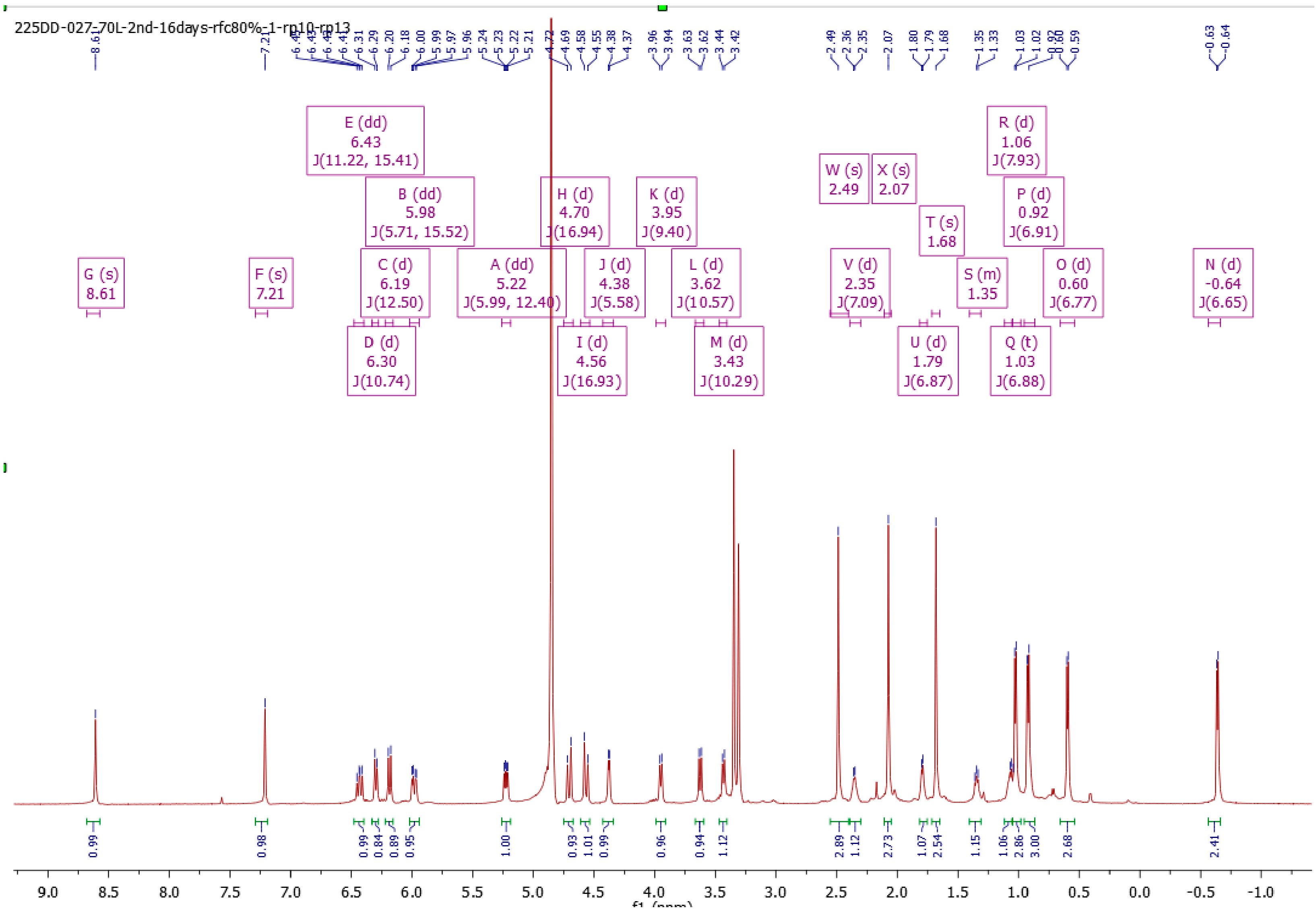

Figure S12. ^{1}H NMR spectrum of **6**.

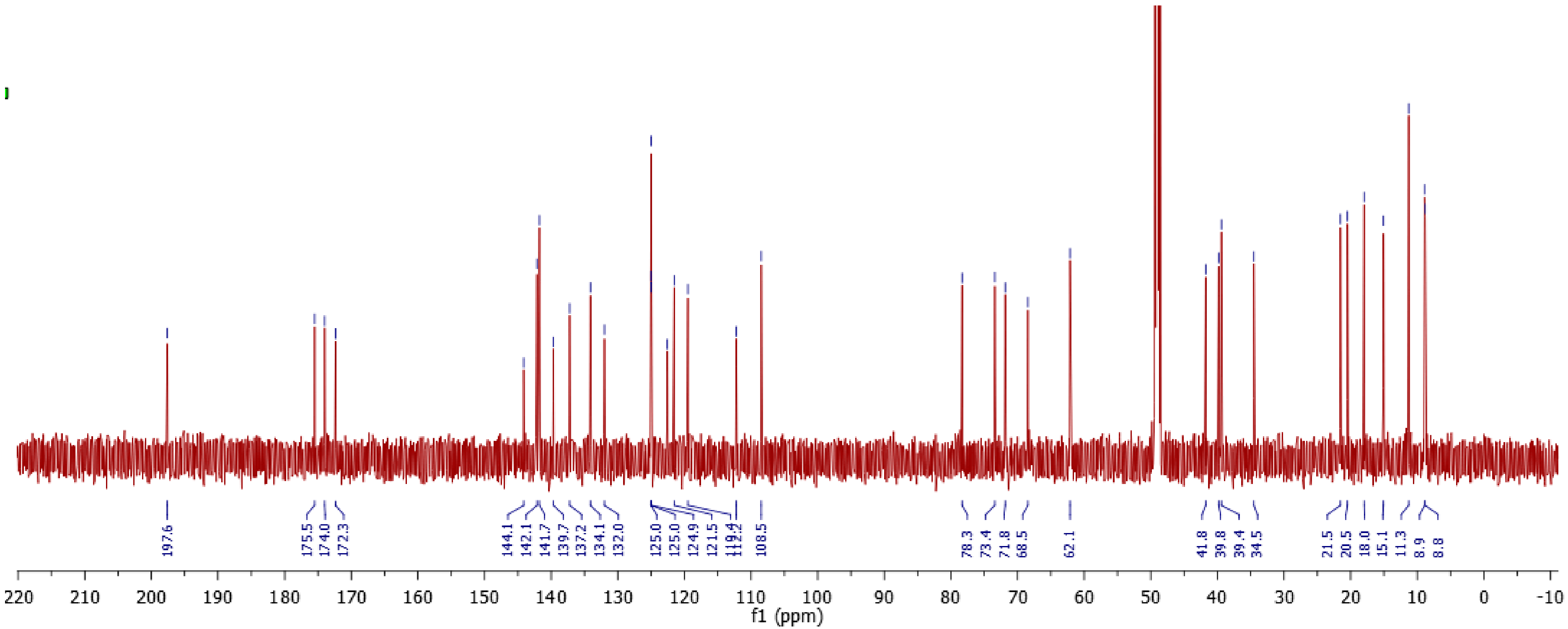

Figure S13. ^{13}C NMR spectrum of **6**.

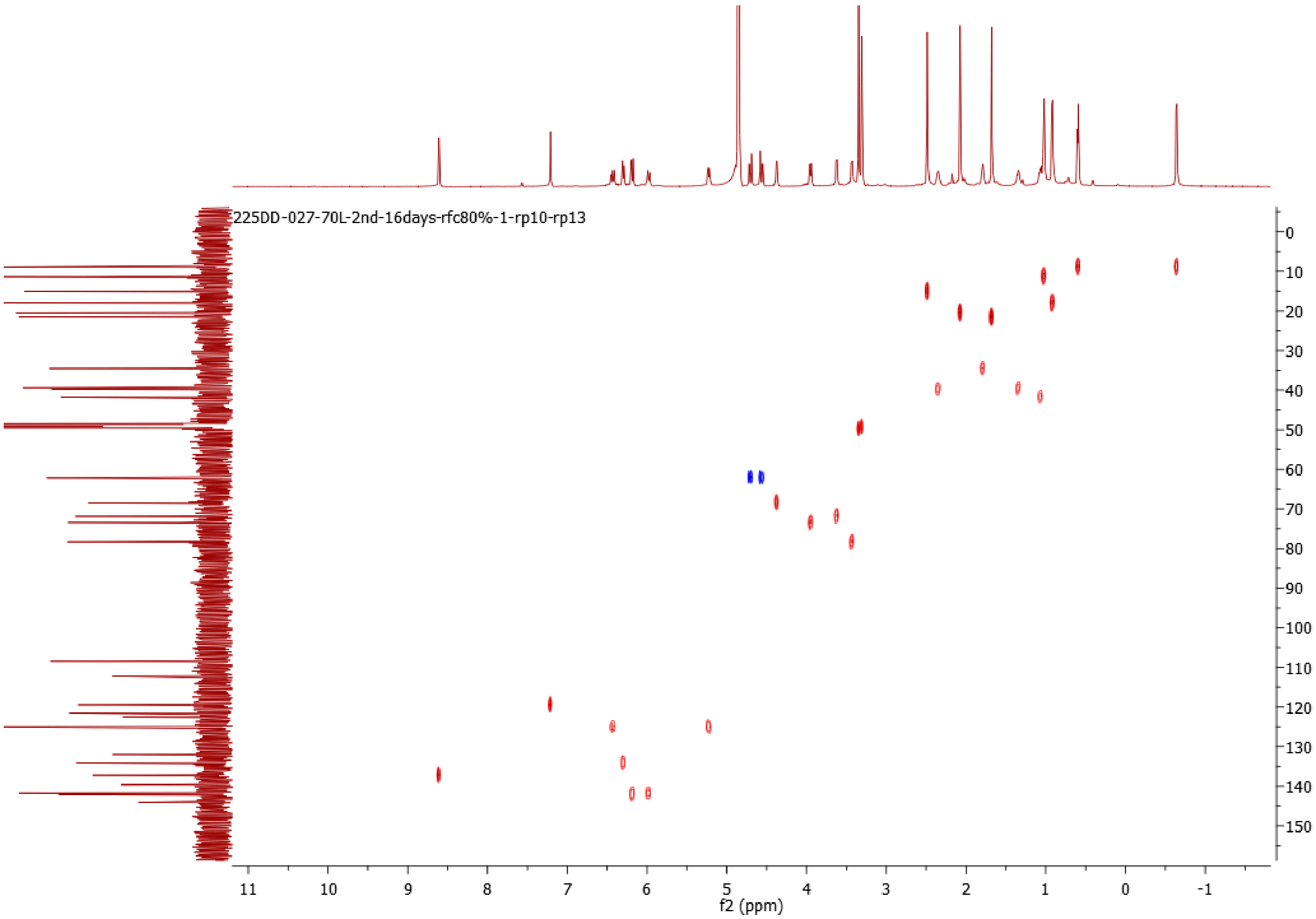

Figure S14. HSQC spectrum of **6**.

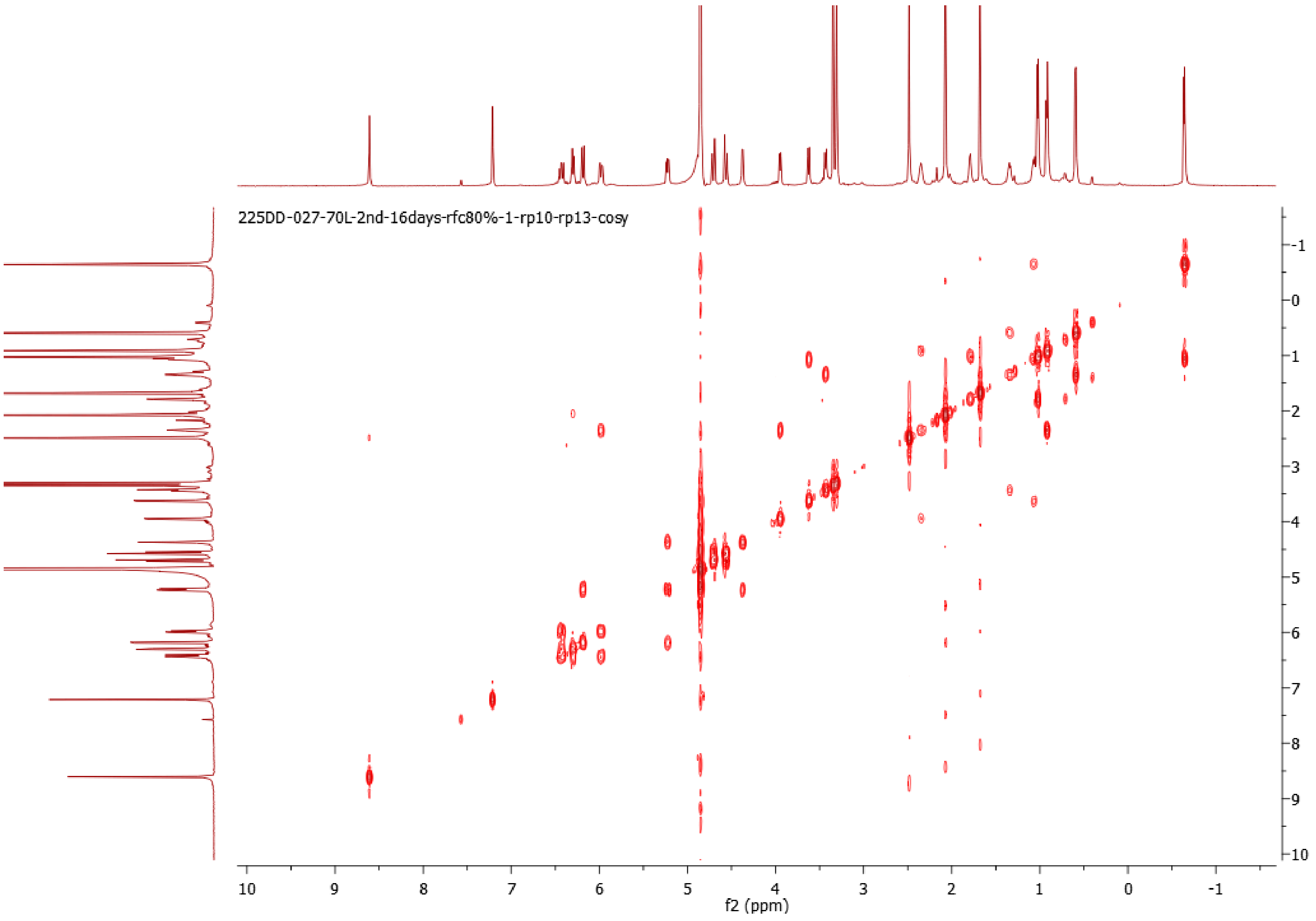

Figure S15. ¹H-¹H COSY spectrum of **6**.

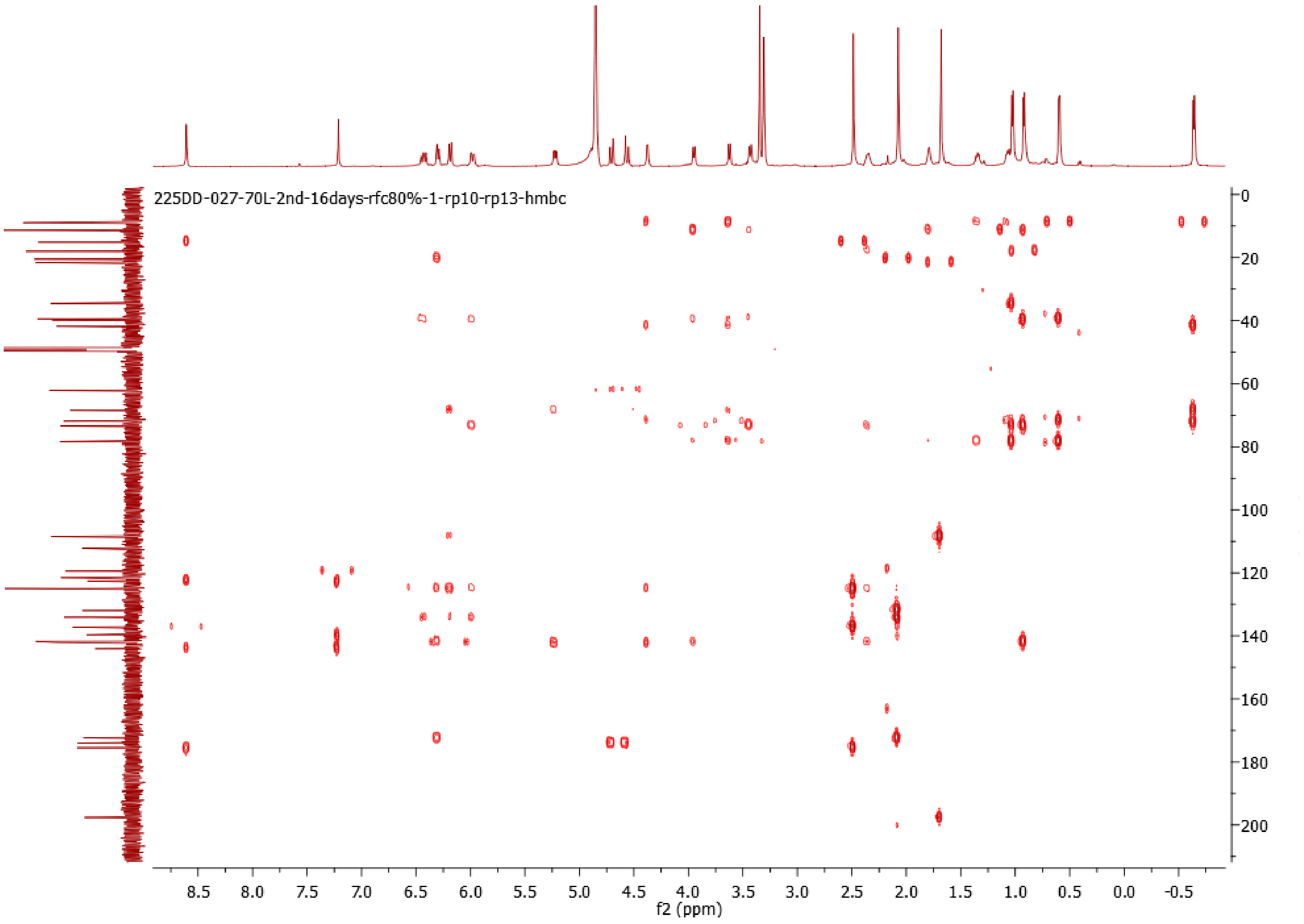

Figure S16. HMBC spectrum of **6**.

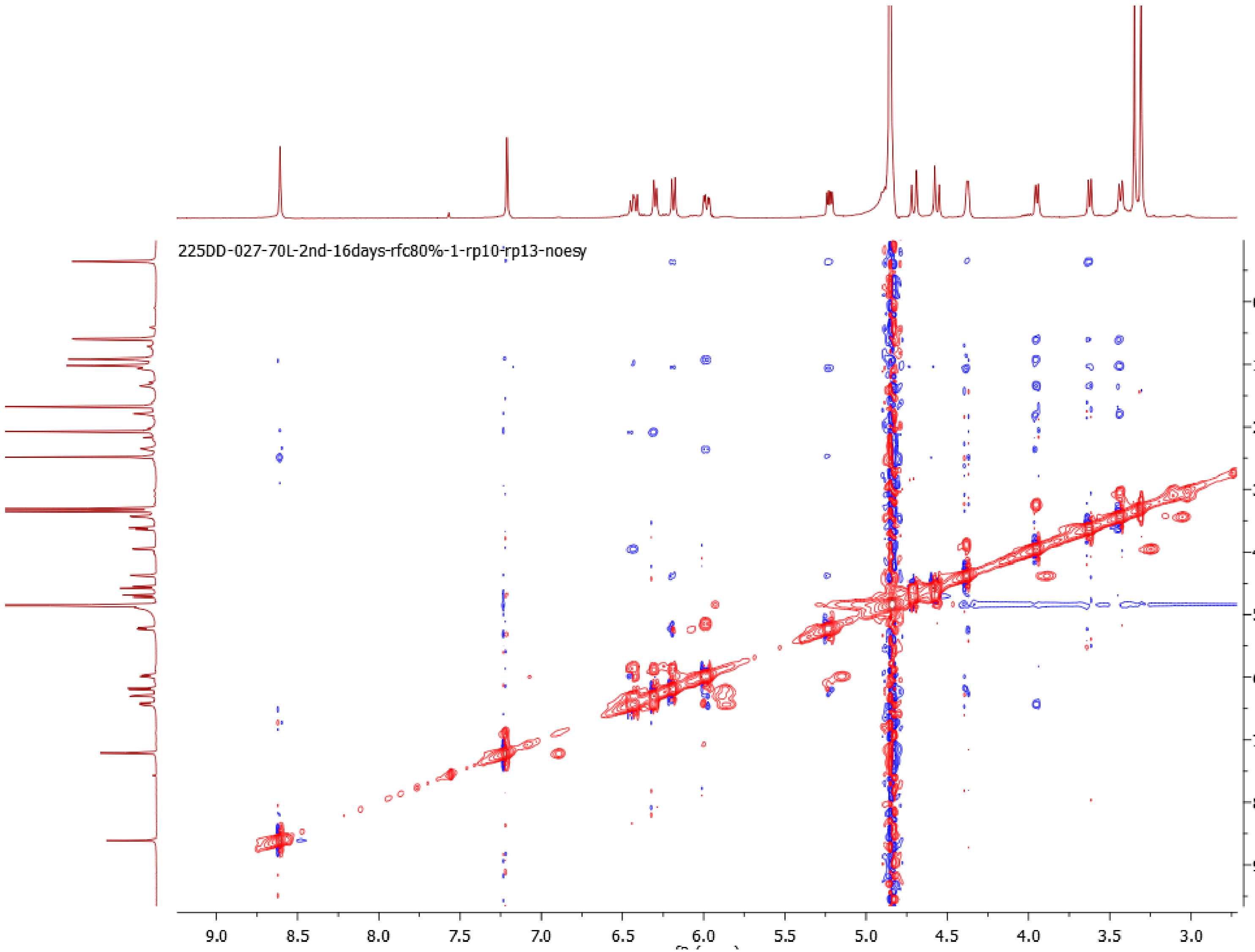

Figure S17. NOESY spectrum of **6**.

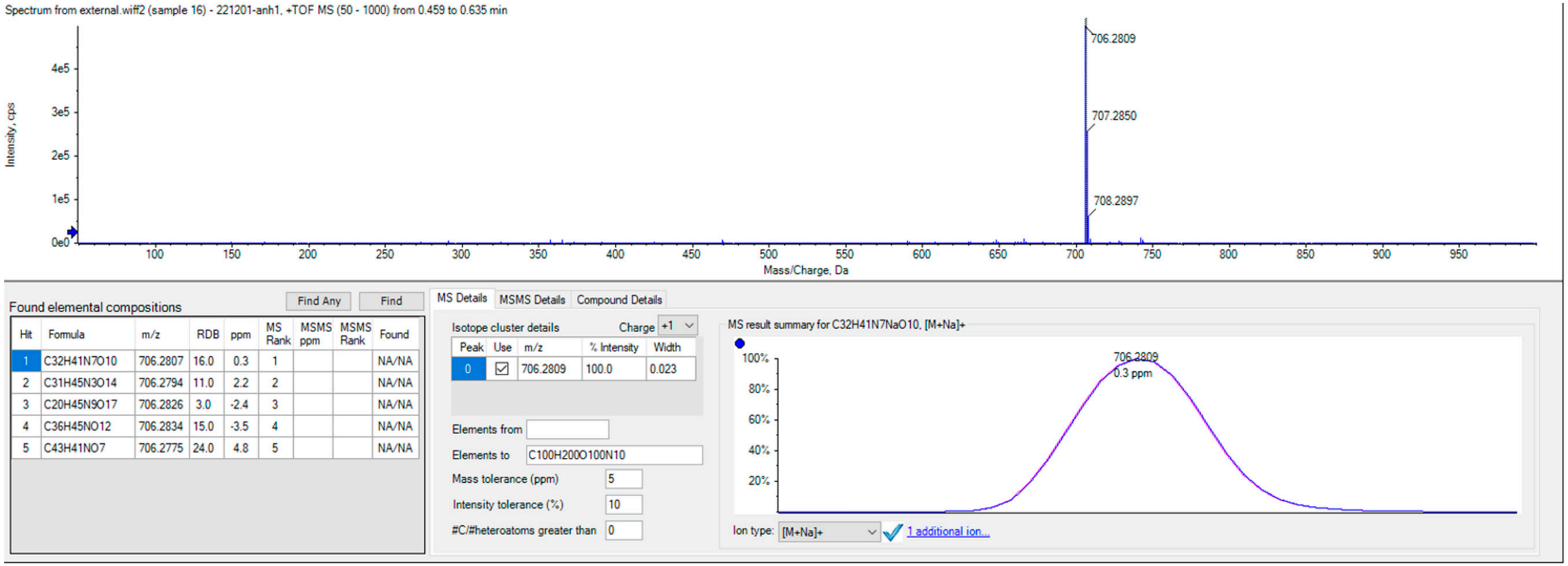

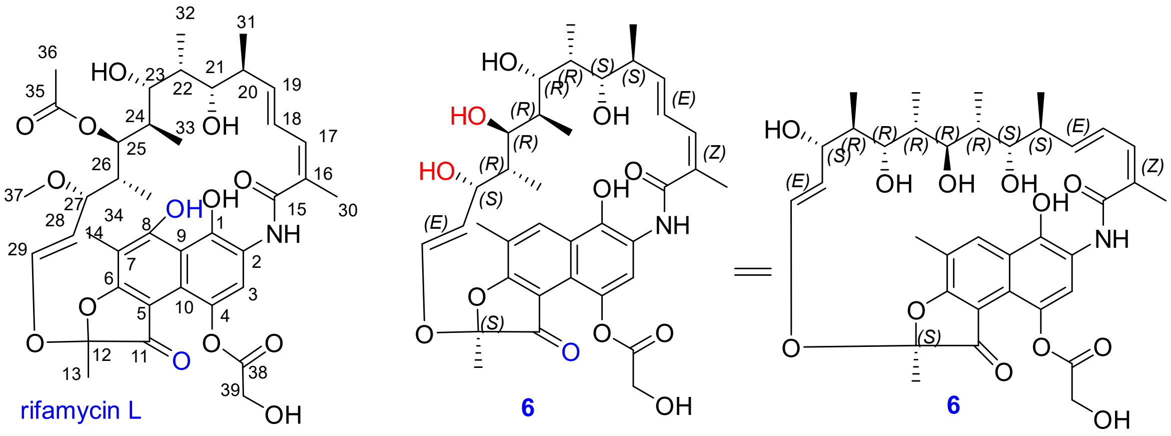

https://doi.org/10.7164/antibiotics.22.369

Figure S18. HRESIMS data of **6**.

Table S2. Comparison of chemical shifts of **7** and **8**.

	Saliniketal A (**8**)		**7**	
No	δ_C	δ_H	δ_H	δ_C
1	175.1, qC			175.1
2	131.4, qC			131.4
3	134.1, CH	6.17, br d (11.1, 1.2)	6.17, br d (11.1)	134.1
4	128.3, CH	6.60, dd (15.3, 11.1)	6.58, dd (15.2, 11.1)	128.3
5	142.0, CH	5.78, dd (15.3, 8.4)	5.78, dd (15.2, 8.3)	142.1
6	42.3, CH	2.35, m (9.3, 8.4, 6.8)	2.34, m	42.3
7	75.8, CH	3.71, dd (9.3, 1.8)	3.73, dd (9.3, 1.3)	75.6
8	35.7, CH	1.88, m (7.4, 4.9, 1.8)	1.89, m	36.3
9	78.2, CH	3.52, dd (8.3, 4.9)	3.50, dd (7.9, 4.6)	78.9
10	37.1, CH	1.84, br dq (8.3, 7.2, 1.4)	1.81, m	38.5

11	74.9, CH	3.97, br d (10.8, 1.4)	4.05, dd (10.2, 1.2)	83.5
12	35.2, CH	2.00, dqd (10.8, 7.3, 3.4)	2.00, m	44.1
13	81.6, CH	4.23, br dd (6.3, 3.4)	3.46, t (8.4)	82.7
14a	24.9, CH_2	1.94, m	3.95, td (8.7, 3.5)	80.8
14b		1.90, m		
15a	35.1, CH_2	2.05, m	2.75 dd (15.6, 3.5)	48.5
15b		1.80, m	2.63 dd (15.6, 8.9)	
16	106.4, qC			210.3
17	24.2, CH_3	1.39, s	2.18, s	30.6
18	20.9, CH_3	1.94, d (1.2)	1.95, s	20.9
19	17.1, CH_3	0.96, d (6.8)	0.95, d (6.9)	17.1
20	11.1, CH_3	1.02, d (7.3)	0.98, d (7.0)	10.9
21	10.2, CH_3	0.89, d (7.2)	0.95, d (6.9)	10.6

22	12.8, CH_3	0.76, d (7.3)	1.05, d (6.5)	14.2
J. Nat. Prod. **2007**, *70*, 1, 83–88				

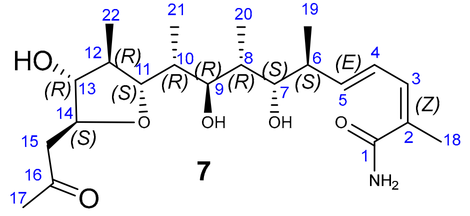

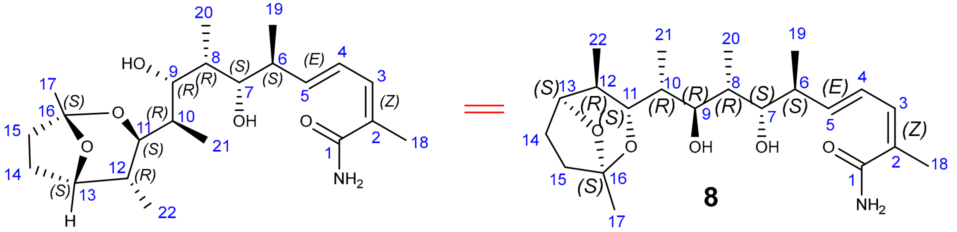

Structure of saliniketal A (**8**) drawn in different ways

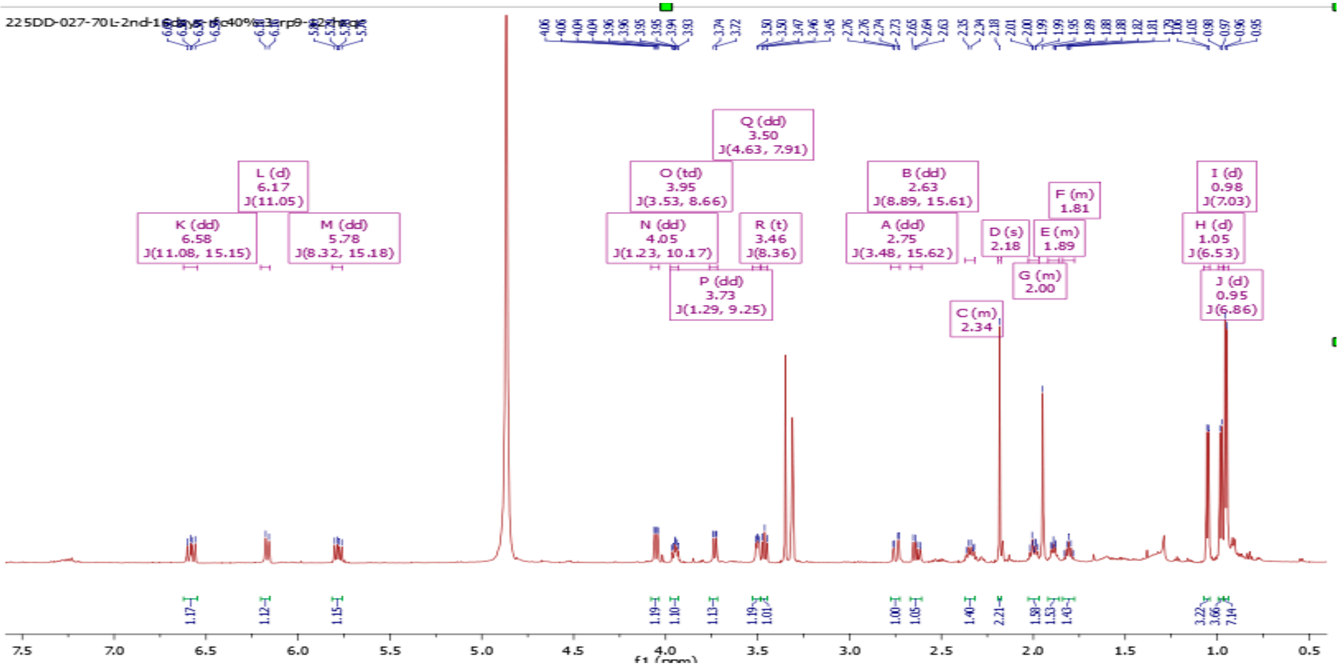

Figure S19. ^{1}H NMR spectrum of **7**.

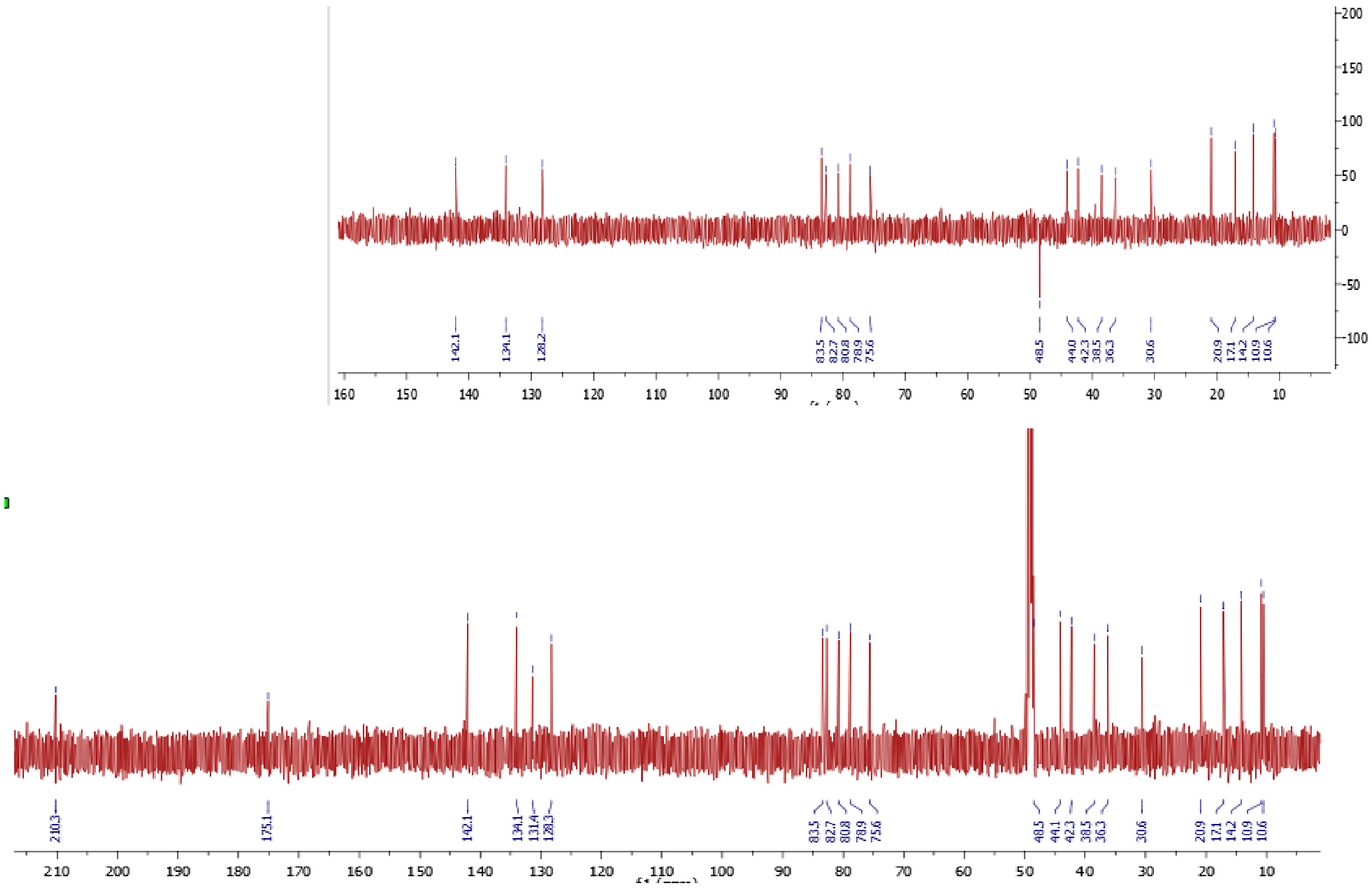

Figure S20. ^{13}C NMR spectrum of **7**.

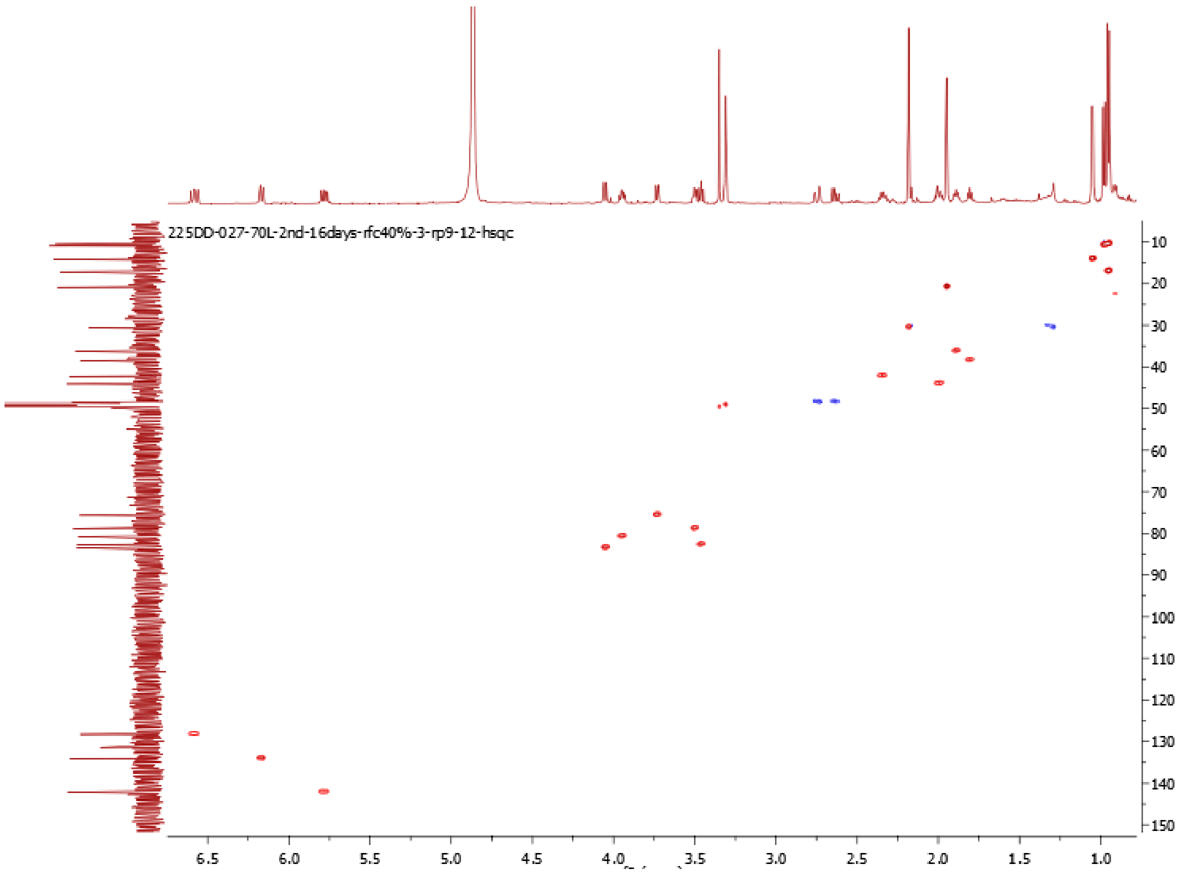

Figure S21. HSQC spectrum of **7**.

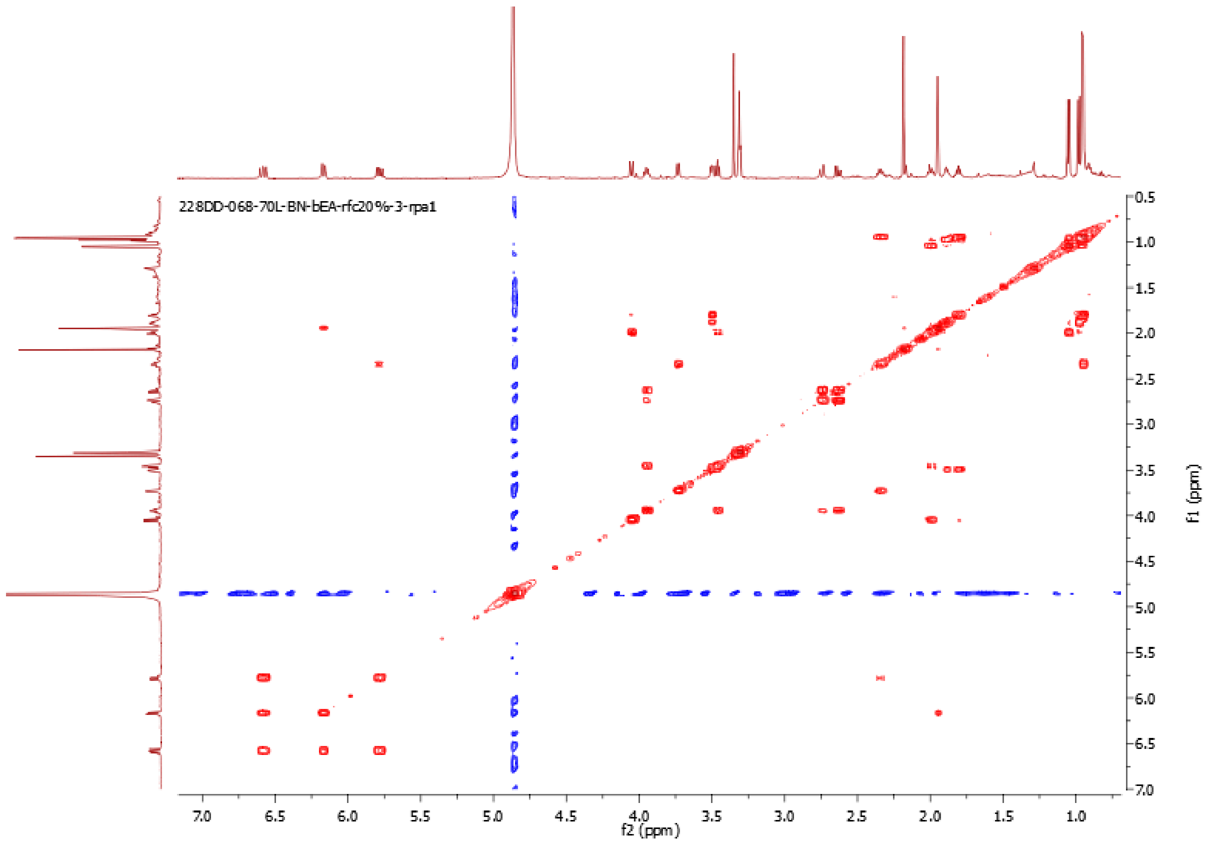

Figure S22. ^{1}H-^{1}H COSY spectrum of **7**.

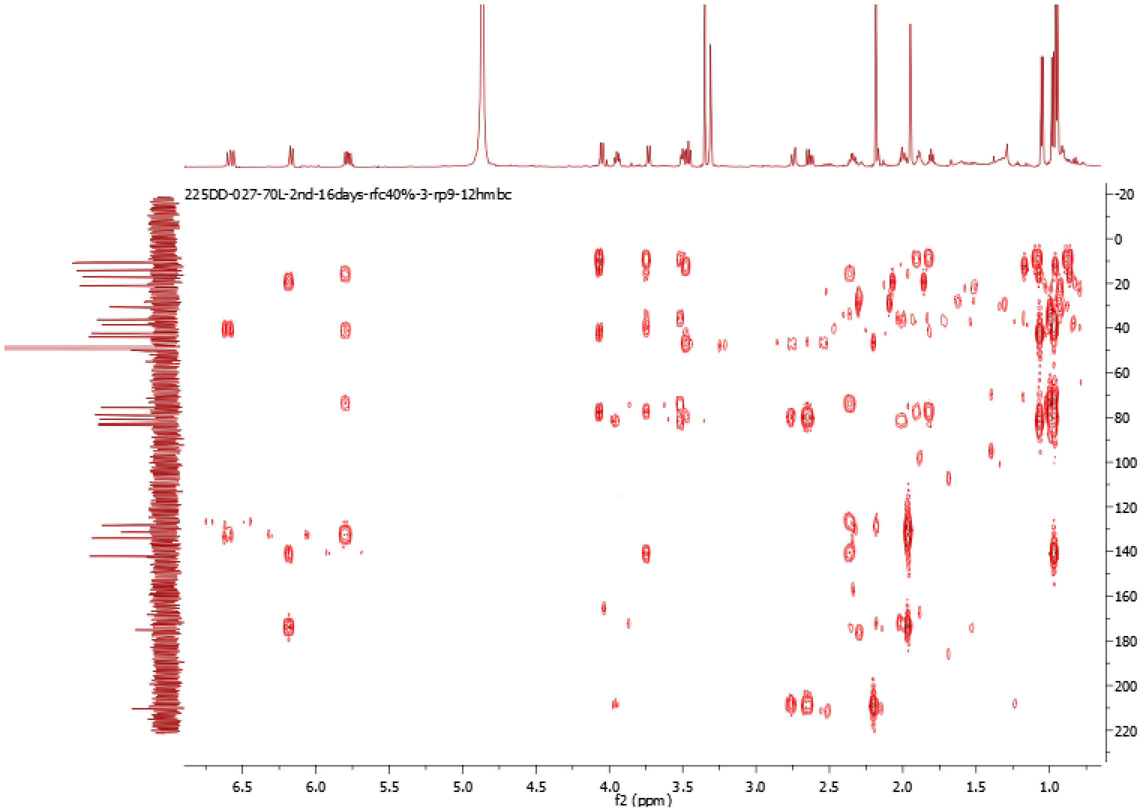

Figure S23. HMBC spectrum of **7**.

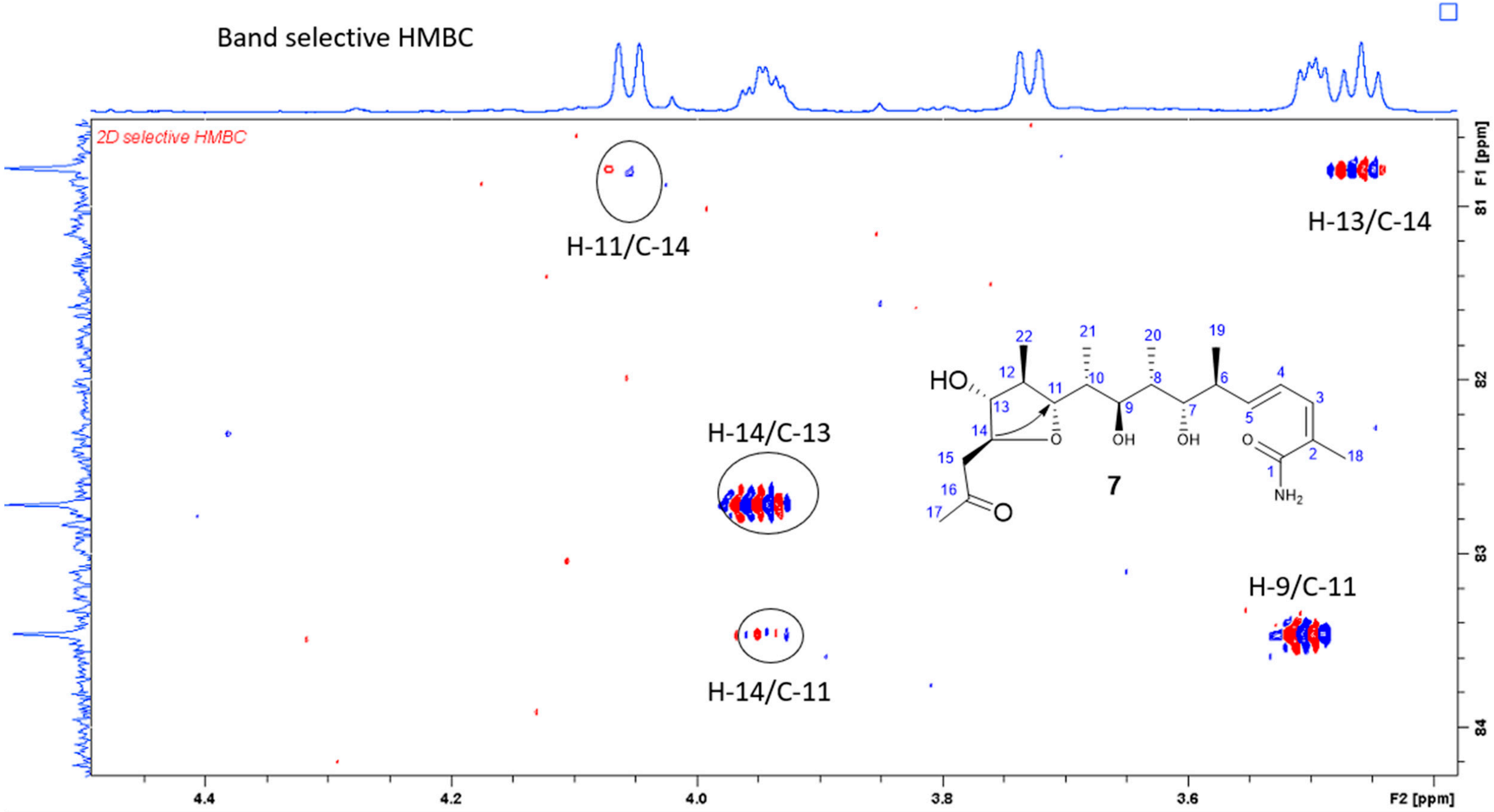

Figure S24. Band selective HMBC of **7**.

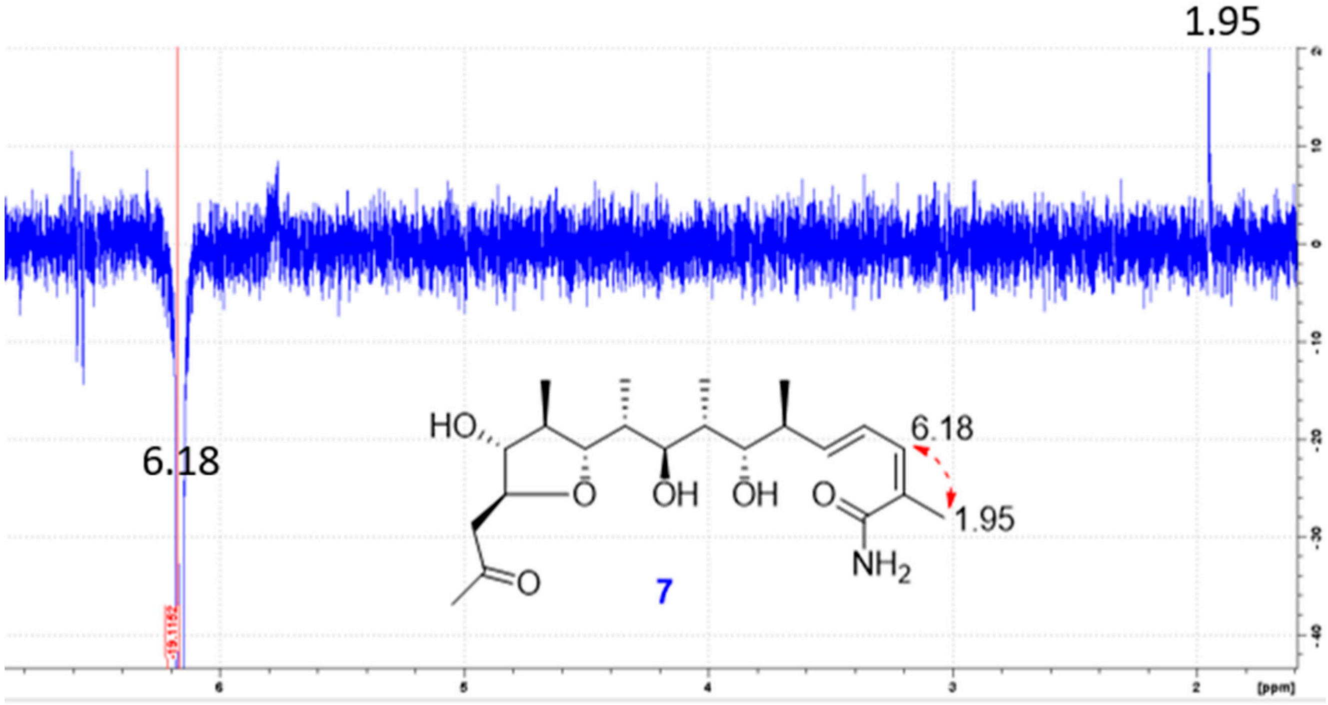

Figure S25. Selective 1D NOESY spectrum of **7** (irradiated at H-3).

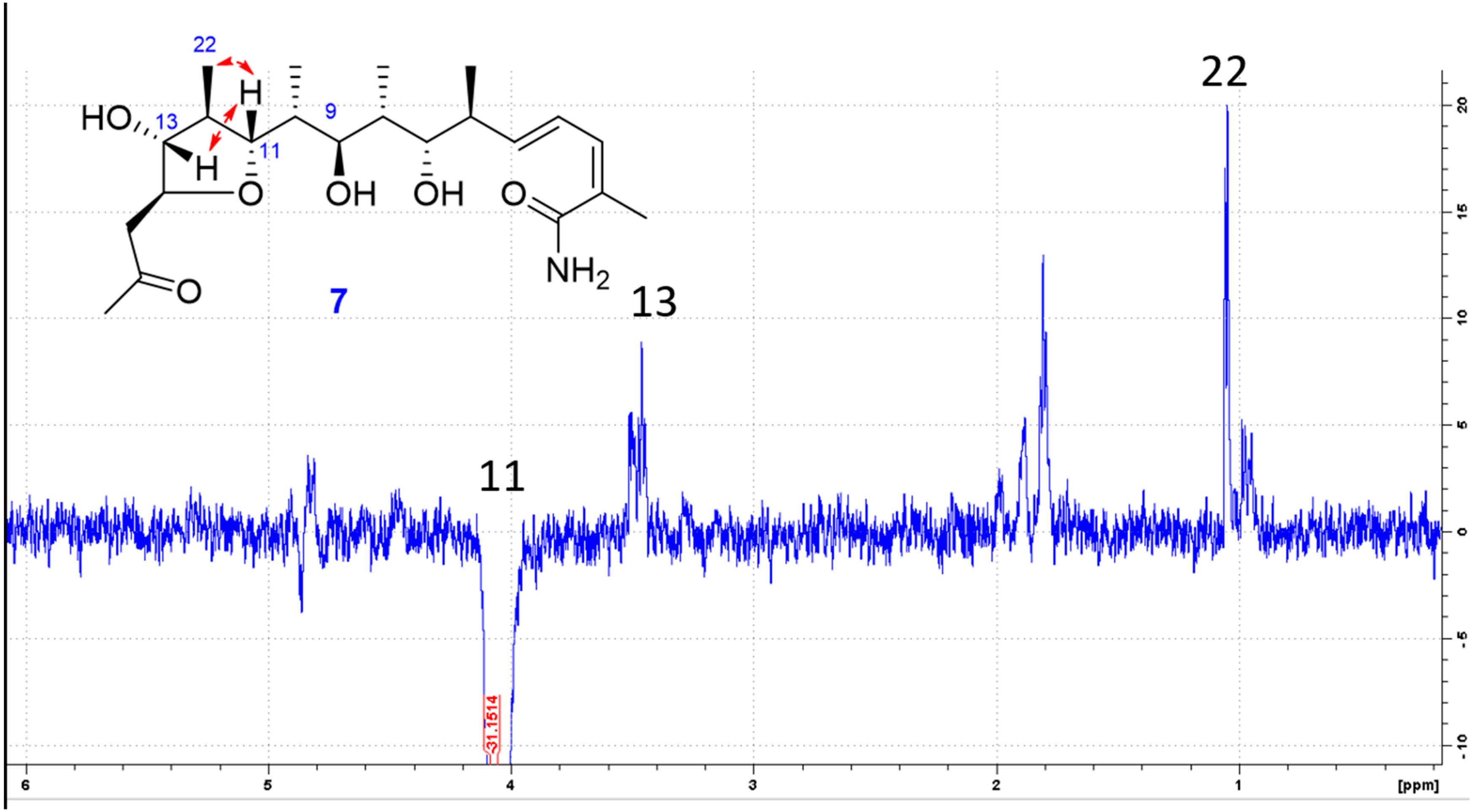

Figure S26. Selective 1D NOESY spectrum of **7** (irradiated at H-11).

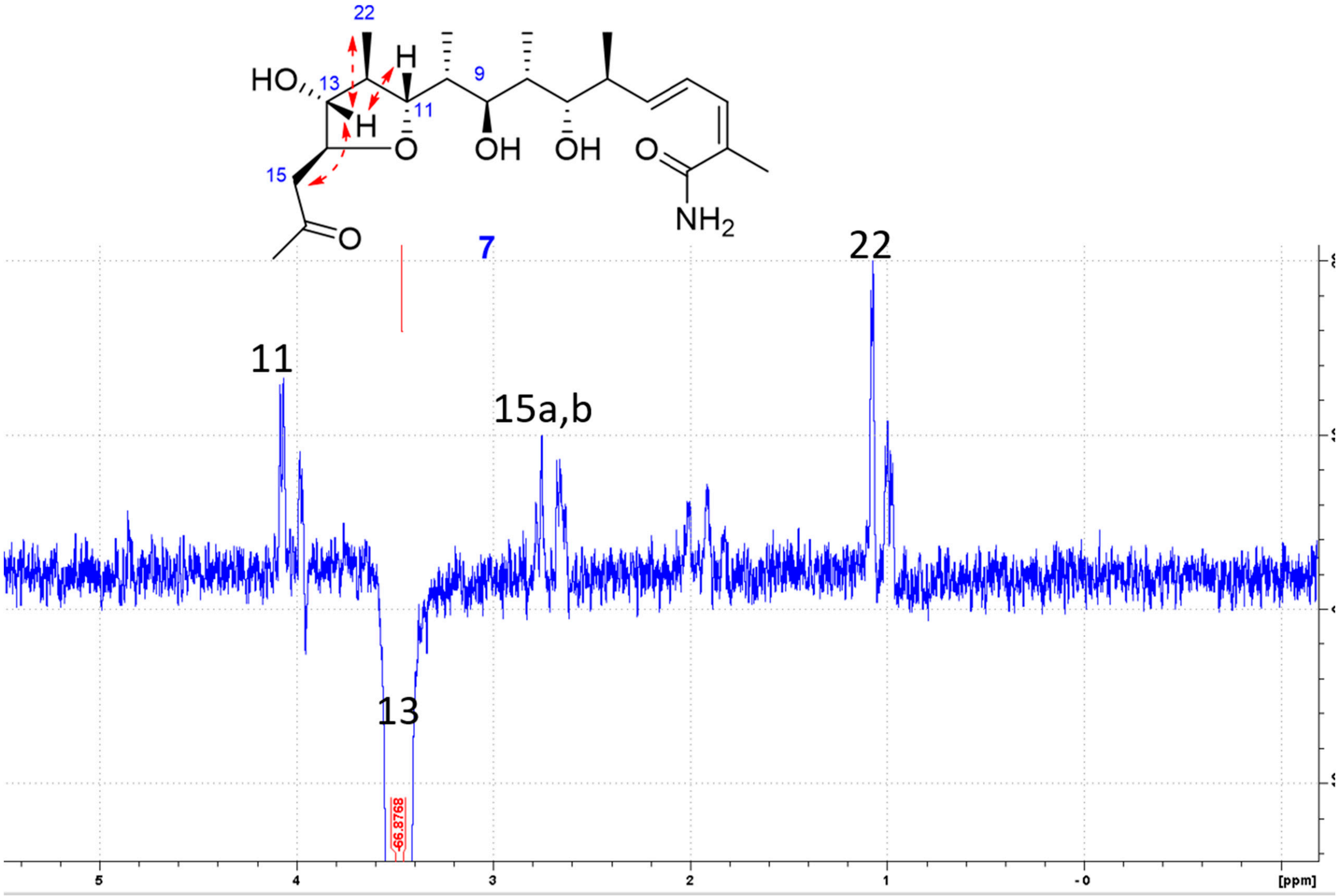

Figure S27. Selective 1D NOESY spectrum of **7** (irradiated at H-13).

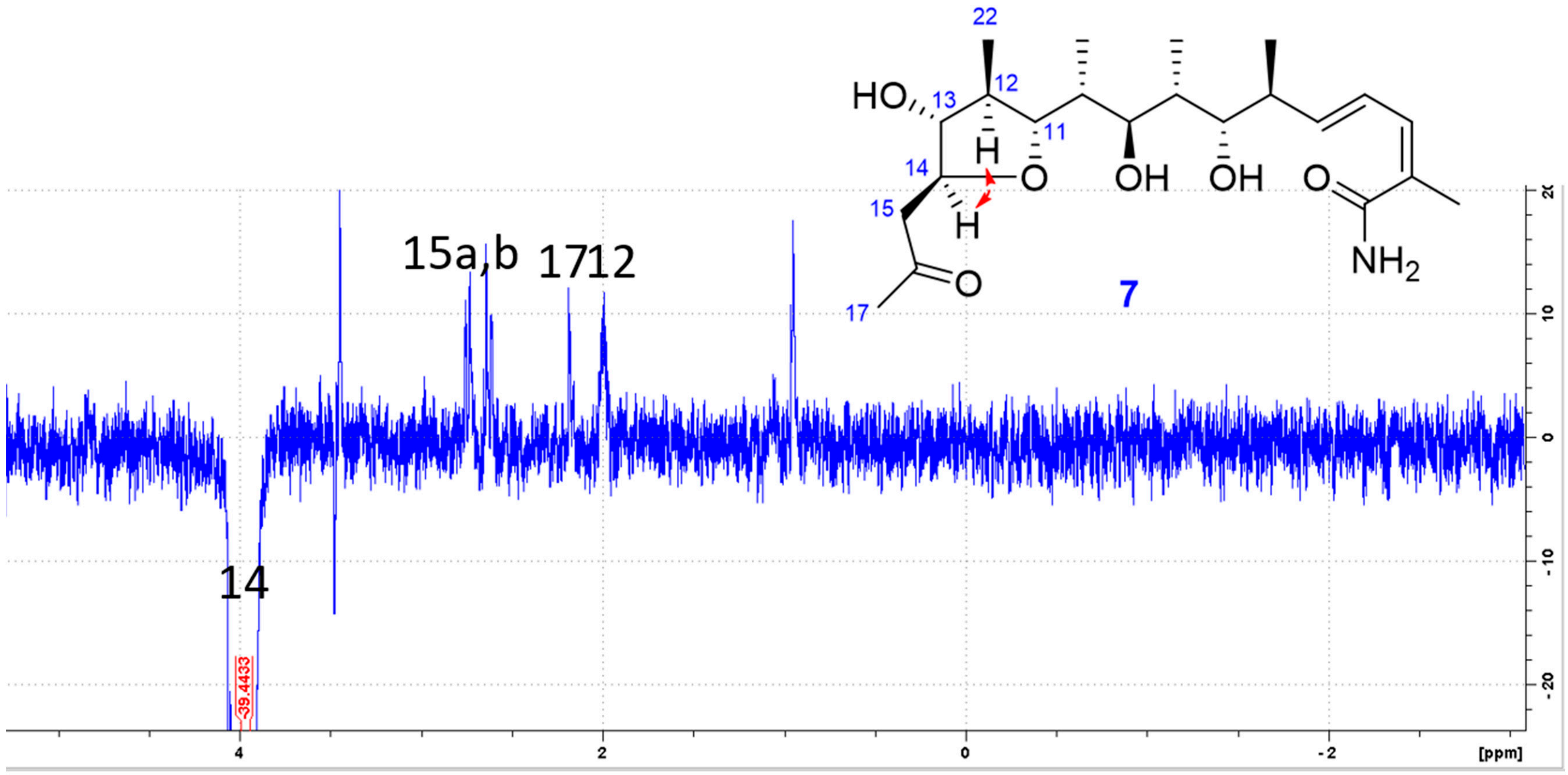

Figure S28. Selective 1D NOESY spectrum of **7** (irradiated at H-14).

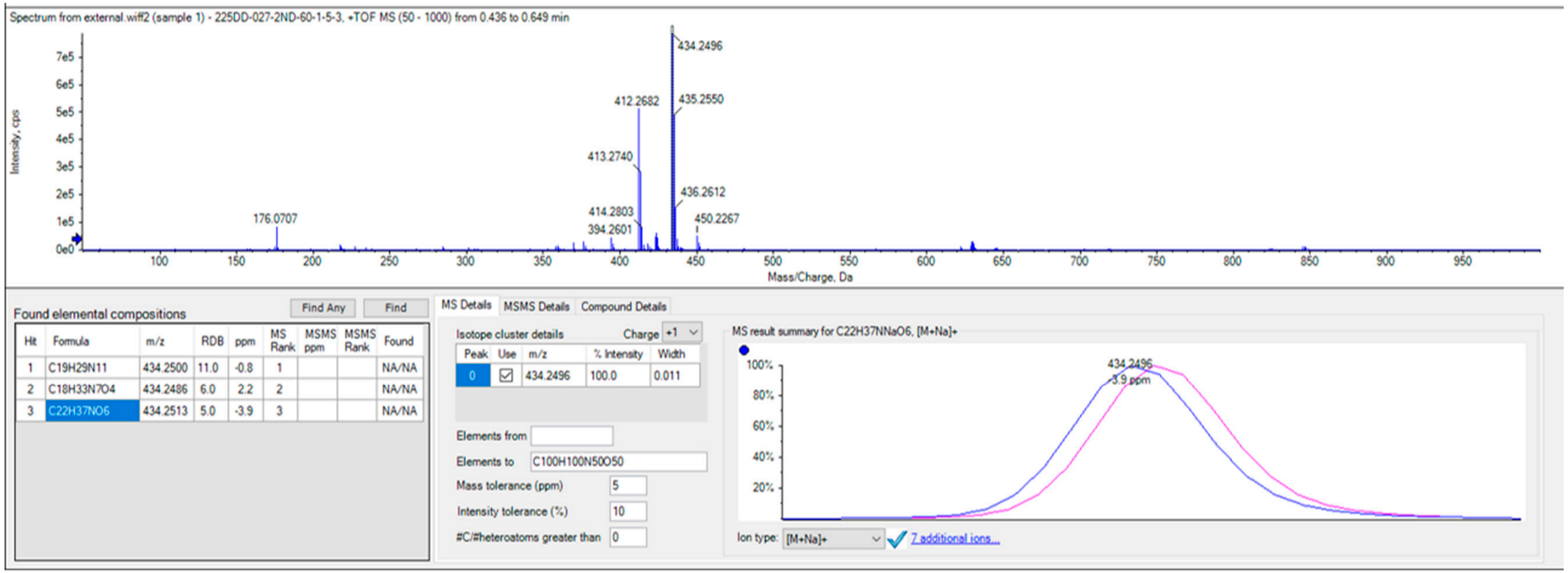

Hit	Formula	m/z	RDB	ppm	MS Rank	MSMS ppm	MSMS Rank	Found
1	C19H29N11	434.2500	11.0	-0.8	1			NA/NA
2	C18H33N7O4	434.2486	6.0	2.2	2			NA/NA
3	C22H37NO6	434.2513	5.0	-3.9	3			NA/NA

Figure S29. HRESIMS data of **7**.

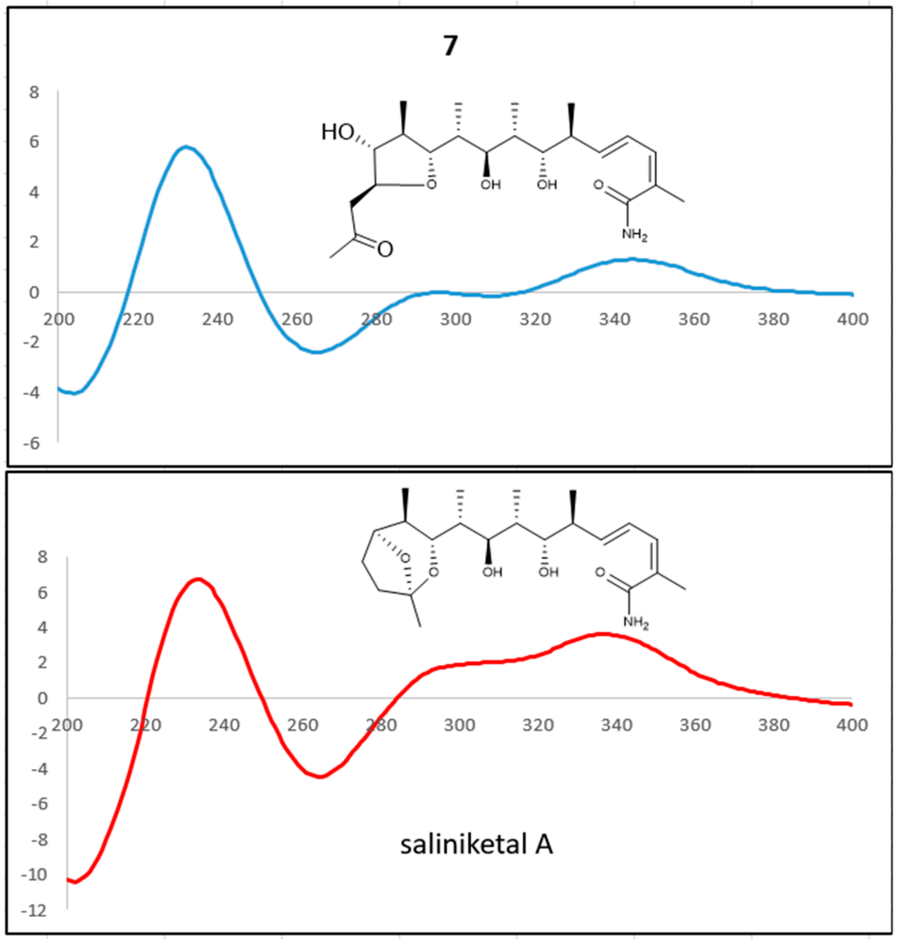

Figure S30. Comparison of ECD spectra of **7** and saliniketal A (**8**).

	ACHN		MDA-MB-231		HCT-15		PC-3		HUGC-3		NCI-H-23	
	1		1		1		1		1		1	
Conc.(ug/ml)	Mean	SD	Mean	SD	Mean	SD	Mean	SD	Mean	SD	Mean	SD
30	-13.46	8.64	-10.07	4.32	-11.13	8.02	-14.02	5.97	-19.37	6.64	-12.90	5.30
10	26.00	2.26	31.39	4.33	27.32	8.81	16.40	6.58	14.23	5.85	17.31	2.11
3	78.78	6.67	79.19	3.07	83.64	4.91	87.18	4.75	80.37	6.25	82.95	5.40
1	95.06	2.45	84.64	1.61	92.23	2.59	89.97	4.10	89.69	3.56	92.41	5.11
0.3	97.49	1.54	93.76	3.48	96.06	8.91	95.96	5.73	92.55	9.68	97.80	4.00
GI50	5.654		5.883		6.188		5.663		4.954		5.386	

	ACHN		MDA-MB-231		HCT-15		PC-3		HUGC-3		NCI-H-23	
	6		6		6		6		6		6	
Conc.(ug/ml)	Mean	SD	Mean	SD	Mean	SD	Mean	SD	Mean	SD	Mean	SD
30	29.55	5.88	34.66	0.97	22.09	6.38	32.58	5.93	29.32	3.65	22.47	5.78
10	88.28	5.86	82.39	1.74	76.73	3.91	72.30	6.97	84.25	5.81	74.24	5.17
3	96.37	1.05	85.81	2.02	84.79	4.55	85.59	8.45	90.44	4.68	77.79	6.95
1	97.29	1.47	89.28	2.86	92.10	6.24	91.40	3.21	95.38	4.52	89.49	3.79
0.3	99.42	1.20	92.83	4.13	97.94	5.71	97.78	6.39	97.61	4.36	98.55	2.98
GI50	21.490		21.400		16.530		18.160		20.320		14.920	

	ACHN		MDA-MB-231		HCT-15		PC-3		HUGC-3		NCI-H-23	
	7		7		7		7		7		7	
Conc.(ug/ml)	Mean	SD	Mean	SD	Mean	SD	Mean	SD	Mean	SD	Mean	SD
30	79.41	6.71	79.78	3.07	72.88	2.97	79.47	4.05	88.30	7.14	77.86	9.12
10	83.99	2.29	85.56	6.09	79.84	6.60	82.70	3.50	89.97	6.94	83.44	1.85
3	88.54	3.57	89.28	3.22	86.79	5.49	88.82	3.01	92.02	8.35	87.30	4.02
1	95.88	4.10	91.42	2.64	90.40	6.92	94.55	1.09	94.12	4.90	91.13	3.09
0.3	98.47	1.70	94.17	4.83	94.93	3.28	98.76	1.90	98.55	6.28	100.24	4.15
GI50	>30		>30		>30		>30		>30		>30	

	ACHN		MDA-MB-231		HCT-15		PC-3		HUGC-3		NCI-H-23	
	8		8		8		8		8		8	
Conc.(ug/ml)	Mean	SD	Mean	SD	Mean	SD	Mean	SD	Mean	SD	Mean	SD
30	80.26	6.78	79.05	5.44	82.57	3.96	78.99	2.29	81.37	6.38	77.78	6.01
10	86.73	5.41	83.58	6.20	86.97	2.07	84.29	5.13	84.62	3.27	81.08	4.17
3	88.73	4.08	87.41	6.25	88.66	7.40	93.69	2.14	88.33	4.19	90.00	5.81
1	91.61	3.69	93.24	4.06	93.93	4.79	95.44	3.10	90.40	6.87	92.95	3.16
0.3	93.50	4.29	98.34	1.89	98.23	4.60	97.19	7.22	98.63	2.06	95.22	3.67
GI50	>30		>30		>30		>30		>30		>30	

	ACHN		MDA-MB-231		HCT-15		PC-3		HUGC-3		NCI-H-23	
	Adriamycin		Adriamycin		Adriamycin		Adriamycin		Adriamycin		Adriamycin	
onc.(ug/ml)	Mean	SD	Mean	SD	Mean	SD	Mean	SD	Mean	SD	Mean	SD
3	-13.13	4.26	-8.64	6.13	-9.58	3.03	-14.29	5.14	-11.86	3.47	-10.30	3.82
1	-4.84	6.22	-4.95	3.59	-6.92	8.80	-5.59	6.05	-7.22	4.40	-5.08	7.60
0.3	25.58	5.44	19.22	3.95	18.41	4.90	19.63	5.72	10.89	4.93	16.13	5.48
0.1	37.47	5.75	38.11	4.01	29.95	6.43	32.60	3.18	25.42	8.11	37.27	6.81
0.03	93.78	7.62	91.89	1.94	91.35	5.00	90.91	3.33	97.32	3.78	89.45	4.32
GI50	0.091		0.086		0.074		0.078		0.074		0.082	

1	HL-60		Raji		K562		RPMI-8402		NALM6		U266		WSU-DLCL2	
(μg/ml)	MEAN	SD	MEAN	SD	MEAN	SD	MEAN	SD	MEAN	SD	MEAN	SD	MEAN	SD
30	0.8	0.1	0.5	0.1	2.1	0.2	0.4	0.1	0.0	0.0	6.5	1.1	0.2	0.0
10	38.9	2.3	8.9	0.7	30.4	2.6	11.5	0.5	7.5	3.3	18.2	1.2	9.0	0.6
3	78.1	6.6	61.6	5.4	67.7	5.0	54.0	4.0	49.8	4.5	40.8	2.3	61.3	1.9
1	88.2	8.0	87.5	7.3	84.8	2.7	85.6	4.2	83.5	2.5	64.7	4.4	60.1	6.6
0.3	93.8	5.2	93.2	6.8	87.9	4.2	98.0	3.2	103.8	4.4	76.1	4.7	62.1	7.3
IC50	6.66		3.59		4.80		3.18		2.86		1.76		1.58	

6	HL-60		Raji		K562		RPMI-8402		NALM6		U266		WSU-DLCL2	
(μg/ml)	MEAN	SD	MEAN	SD	MEAN	SD	MEAN	SD	MEAN	SD	MEAN	SD	MEAN	SD
30	0.0	0.0	0.0	0.0	0.0	0.0	0.0	0.0	0.0	0.0	0.0	0.0	0.0	0.0
10	0.0	0.0	0.5	0.2	34.0	6.4	0.0	0.0	0.0	0.0	0.0	0.0	0.1	0.0
3	60.1	1.1	123.3	3.3	79.5	3.0	22.1	2.7	58.3	2.1	23.8	5.2	68.8	2.5
1	98.8	7.0	127.7	2.2	94.1	3.8	75.9	5.7	86.9	4.9	73.7	4.5	91.7	2.4
0.3	104.4	6.0	109.5	5.9	95.7	3.6	92.8	3.6	87.2	9.8	81.1	9.6	88.6	2.4
IC50	3.27		8.23		6.51		1.67		3.25		1.57		3.69	

7	HL-60		Raji		K562		RPMI-8402		NALM6		U266		WSU-DLCL2	
(μg/ml)	MEAN	SD	MEAN	SD	MEAN	SD	MEAN	SD	MEAN	SD	MEAN	SD	MEAN	SD
30	60.3	6.0	179.9	19.8	86.8	3.0	47.7	5.5	6.5	7.9	74.0	2.5	63.1	3.0
10	101.9	5.9	249.6	15.2	96.2	2.1	90.1	3.2	56.7	5.2	95.0	3.8	114.6	3.9
3	102.2	4.7	191.0	5.6	97.4	2.7	100.2	3.3	104.1	4.8	100.9	5.9	111.8	3.9
1	102.4	3.5	126.7	12.2	95.8	2.1	95.2	4.3	99.5	3.1	94.6	1.2	98.0	0.7
0.3	97.3	3.8	91.4	6.4	94.2	2.2	92.4	7.4	93.7	7.7	94.6	9.1	91.5	5.9
IC50	>30		>30		>30		28.70		11.00		>30		>30	

8	HL-60		Raji		K562		RPMI-8402		NALM6		U266		WSU-DLCL2	
(μg/ml)	MEAN	SD	MEAN	SD	MEAN	SD	MEAN	SD	MEAN	SD	MEAN	SD	MEAN	SD
30	87.9	1.7	137.3	4.1	81.2	1.6	89.4	4.0	86.6	6.6	87.2	1.3	120.6	2.8
10	94.8	5.6	140.1	5.1	96.8	1.8	102.3	6.4	101.3	3.4	93.0	5.2	135.0	6.2
3	95.0	2.2	124.0	9.3	101.6	3.7	109.5	2.8	102.0	4.6	101.2	6.7	117.9	1.8
1	97.2	2.1	116.1	4.9	105.0	2.8	105.0	7.3	104.0	4.6	92.6	3.9	112.5	4.5
0.3	95.7	3.2	92.7	3.1	102.3	2.2	104.3	5.3	102.6	2.8	94.4	4.9	104.8	1.9
IC50	>30		>30		>30		>30		>30		>30		>30	

Adr.	HL-60		Raji		K562		RPMI-8402		NALM6		U266		WSU-DLCL2	
(μM)	MEAN	SD	MEAN	SD	MEAN	SD	MEAN	SD	MEAN	SD	MEAN	SD	MEAN	SD
1					4.3	0.3								
0.3					7.3	0.5								
0.1	1.9	0.2	0.2	0.1	46.8	1.9	0.7	0.1	0.0	0.0	9.6	0.7	0.3	0.1
0.03	35.9	2.0	2.4	0.7	65.9	3.7	8.2	1.4	0.0	0.0	57.4	2.6	4.5	0.4
0.01	77.8	4.8	32.8	1.3	76.3	2.9	71.4	4.1	0.1	0.0	90.1	3.2	10.4	1.1
0.003	96.8	8.6	50.5	5.4			88.8	5.8	43.5	3.4	86.9	8.4	42.7	4.1
0.001	95.7	8.8	62.9	5.3			103.6	7.4	80.1	2.9	85.9	2.3	60.5	4.9
IC50	0.021		0.003		0.057		0.014		0.002		0.034		0.002	

RPMI-1788	Doxorubicin		RPMI-1788	**6**		**1**	
(μM)	MEAN	SD	(μg/ml)	MEAN	SD	MEAN	SD
0.3	0.8	0.1	30	0.0	0.0	0.4	0.0
0.1	6.8	0.6	10	0.2	0.0	20.5	0.7
0.03	29.4	1.2	3	74.4	1.7	81.1	2.7
0.01	55.7	3.1	1	101.9	5.4	109.0	2.8
0.003	92.2	5.8	0.3	100.6	9.2	112.5	4.9
IC50	0.014		IC50	3.366		5.669	
			IC50 μM	4.9283		8.4738	

	**1 (μM)**	**1 (μg/ml)**	**6 (μM)**	**6 (μg/ml)**	**7 (μM)**	**7 (μg/ml)**	**8 (μM)**	**8 (μg/ml)**	**Adr. (μM)**	**Adr. (μg/ml)**
ACHN	8.45	5.654	> 30	21.490	> 30	> 30	> 30	> 30	0.168	0.091
MDA-MB-231	8.79	5.883	> 30	21.400	> 30	> 30	> 30	> 30	0.159	0.086
HCT-15	9.25	6.188	24.2	16.530	> 30	> 30	> 30	> 30	0.137	0.074
PC-3	8.46	5.663	26.59	18.160	> 30	> 30	> 30	> 30	0.144	0.078
NUGC-3	7.41	4.954	29.75	20.320	> 30	> 30	> 30	> 30	0.136	0.074
NCI-H23	8.05	5.386	21.84	14.920	> 30	> 30	> 30	> 30	0.151	0.082
HL-60	9.96	6.66	4.79	3.27	> 30	> 30	> 30	> 30	0.021	0.011
Raji	5.37	3.59	12.05	8.23	> 30	> 30	> 30	> 30	0.003	0.002
K562	7.17	4.80	9.53	6.51	> 30	> 30	> 30	> 30	0.057	0.031
RPMI-8402	4.75	3.18	2.45	1.67	> 30	28.70	> 30	> 30	0.014	0.008
NALM6	4.28	2.86	4.76	3.25	26.76	11.00	> 30	> 30	0.002	0.001
U266	2.63	1.76	2.30	1.57	> 30	> 30	> 30	> 30	0.034	0.018
WSU-DLCL2	2.36	1.58	5.40	3.69	> 30	> 30	> 30	> 30	0.002	0.001
RPMI-1788	8.47	5.67	4.93	3.37	nt	nt	nt	nt	0.014	0.008

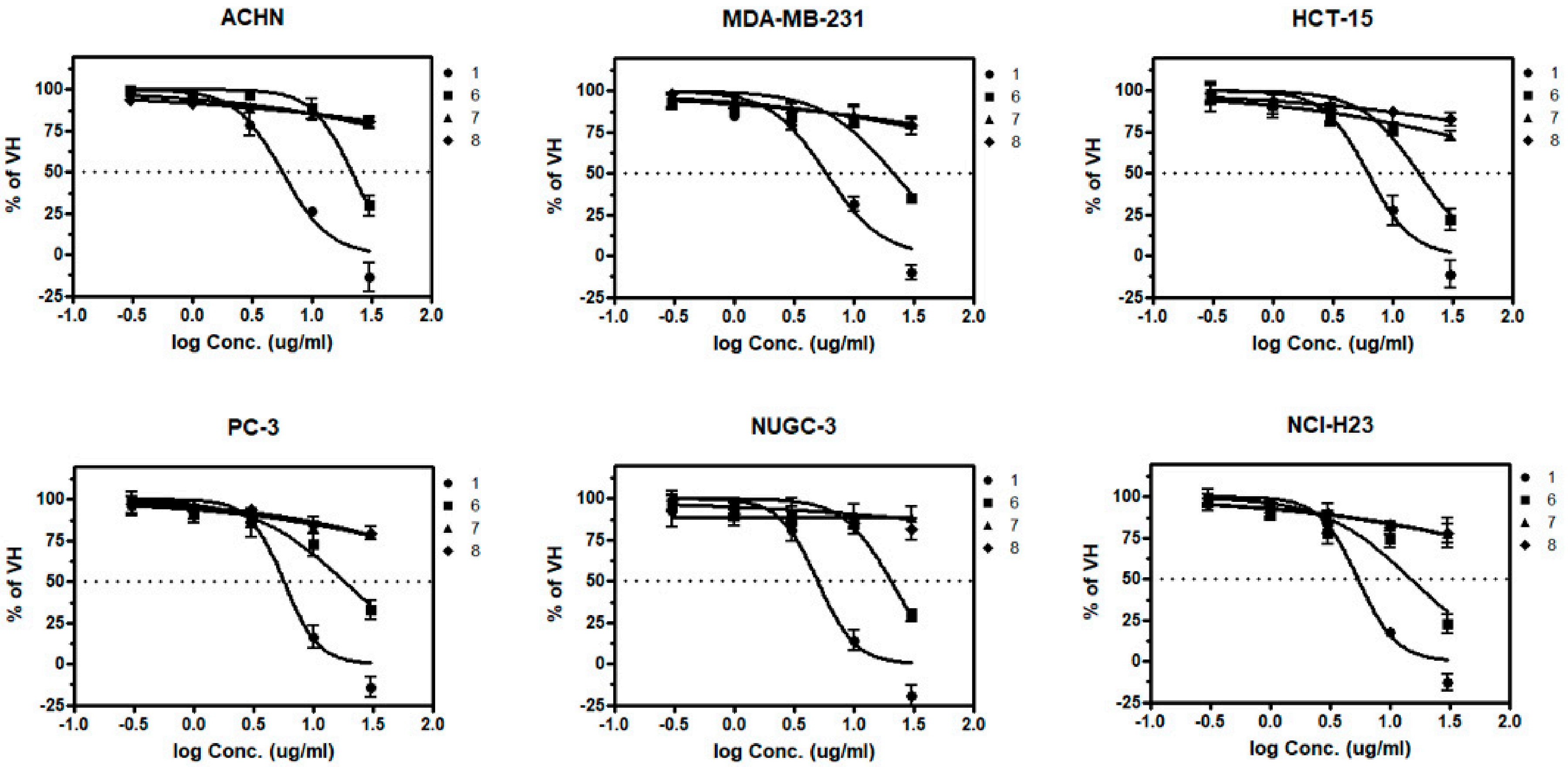
ACHN
MDA-MB-231
HCT-15
PC-3
NUGC-3
NCI-H23
% of VH
log Conc. (ug/ml)
1
6
7
8

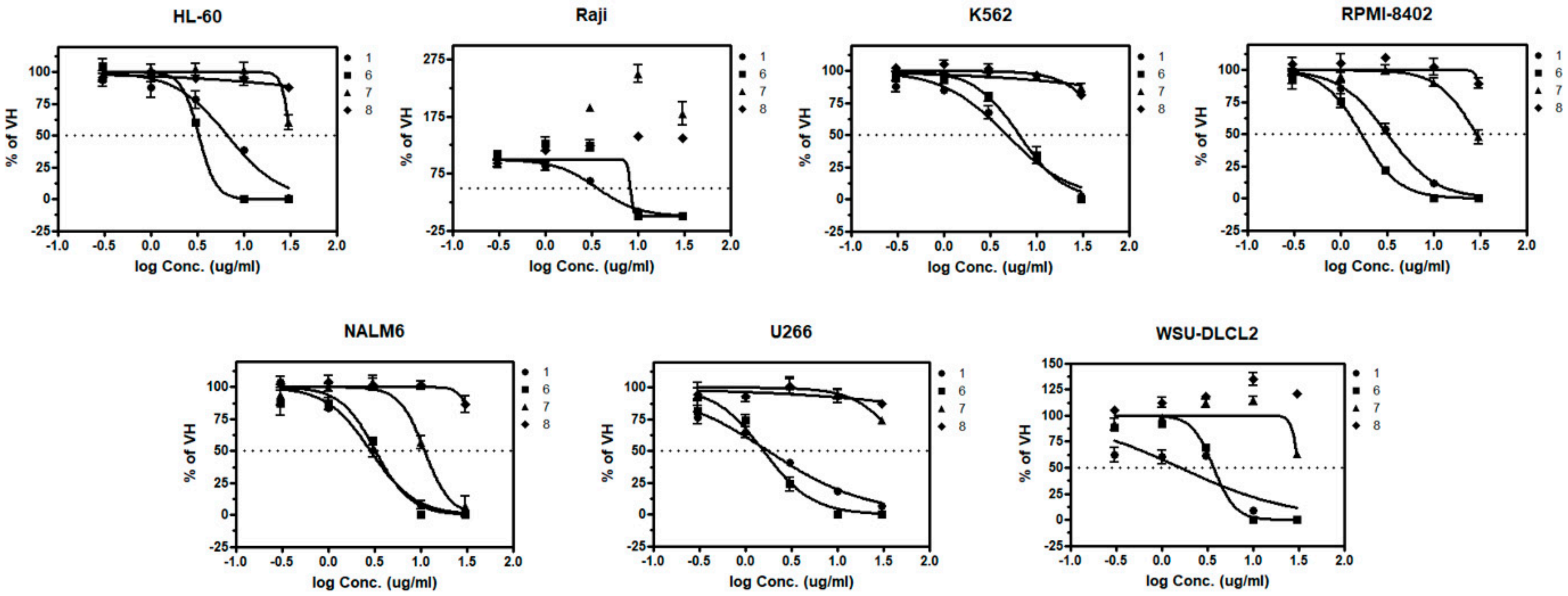

Figure S31. Results of cytotoxicity test and dose-response curves for compounds **1** and **6-8.**
